# Bimetallic Activation of SF_6_ by a Bis(Gallylene)

**DOI:** 10.1021/acs.inorgchem.5c04924

**Published:** 2025-12-08

**Authors:** Aylin Nagel, Max Neubauer, Douglas L. Miller, Phil Köhler, Ralf Tonner-Zech, Robert Kretschmer

**Affiliations:** † Institut für Chemie, 38869 Technische Universität Chemnitz, Strasse der Nationen 62, Chemnitz 09111, Germany; ‡ Wilhelm-Ostwald-Institut für Physikalische Und Theoretische Chemie, 200999 Universität Leipzig, Linnéstr. 2, Leipzig 04103, Germany; § Institut für Anorganische und Analytische Chemie, Friedrich-Schiller-Universität Jena, Humboldtstraße 8, Jena 07443, Germany; ∥ Jena Center of Soft Matter, Friedrich-Schiller-Universität Jena, Philosophenweg 7, Jena 07443, Germany

## Abstract

The degradation of the kinetically inert and potent greenhouse gas sulfur hexafluoride by a bimetallic main-group ambiphile (Lewis amphotere) is reported. Reaction of the bis­(gallylene) **1** with SF_6_ at 80 °C results in the selective formation of the bimetallic gallium­(II) fluoride **2**. Irradiation with UV light or the addition of 4-dimethylaminopyridine (4-DMAP) accelerates the reaction, enabling it to proceed already at room temperature. Computations reveal a bimetallic activation scenario and shed light on the accelerating effect of 4-DMAP.

## Graphical abstract

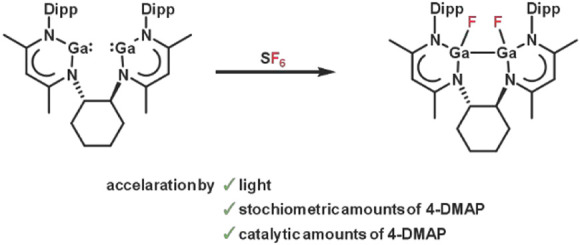

## Introduction

The activation of small molecules constitutes an important and vibrant research area within molecular synthetic chemistry as it possibly provides access to new synthetic methodology utilizing these compounds. Kinetically inert molecules are particularly interesting targets, but their activation remains a challenge. The degradation and utilization of sulfur hexafluoride (SF_6_) received increasing attention in recent years because it has been widely used as an insulating gas in electrical equipment^1^ before its use was restricted due to its a global warming potential, which is significantly higher than that of carbon dioxide.^2^ In order to facilitate a save disposal of SF_6_, approaches on its controlled degradation down to less problematic compounds are needed.^3^ At the same time, the utilization of SF_6_ as either a fluorination agent or as a SF_5_ source^4^ sparked some interest. Following pioneering studies on the reduction of SF_6_ with elemental sodium in the 1950s and 1960s,^5^ various approaches have been established ranging from stoichiometric to catalytic reactions. Research on low-valent transition-metal complexes^6^ including Cr,^7^ Fe,^7^ Ir,^8^ Ni,^9^ Pt,^10^ Rh,^11^ Ti,^12^ V,^7^ and Zr^12^ has dominated the field but in the past decade metal-free approaches^4,13^ as well as methods involving main-group^14^ and rare-earth elements^15^ started to arise. Predominantly, SF_6_ is activated across one active center and electrochemical^3^ and photochemical^13e^ transformations have been reported as well, Figure 1. Herein we report an alternative approach, which combines previous reports on the bimetallic activation by transition metals^11a^ with recent reports on the activation of SF_6_ by low-valent main-group element compounds,^14a,14c^ which allows the activation of sulfur hexafluoride across two main-group metal centers by utilizing the previously reported bis­(gallylene) **1**.^16^

**Figure 1:**
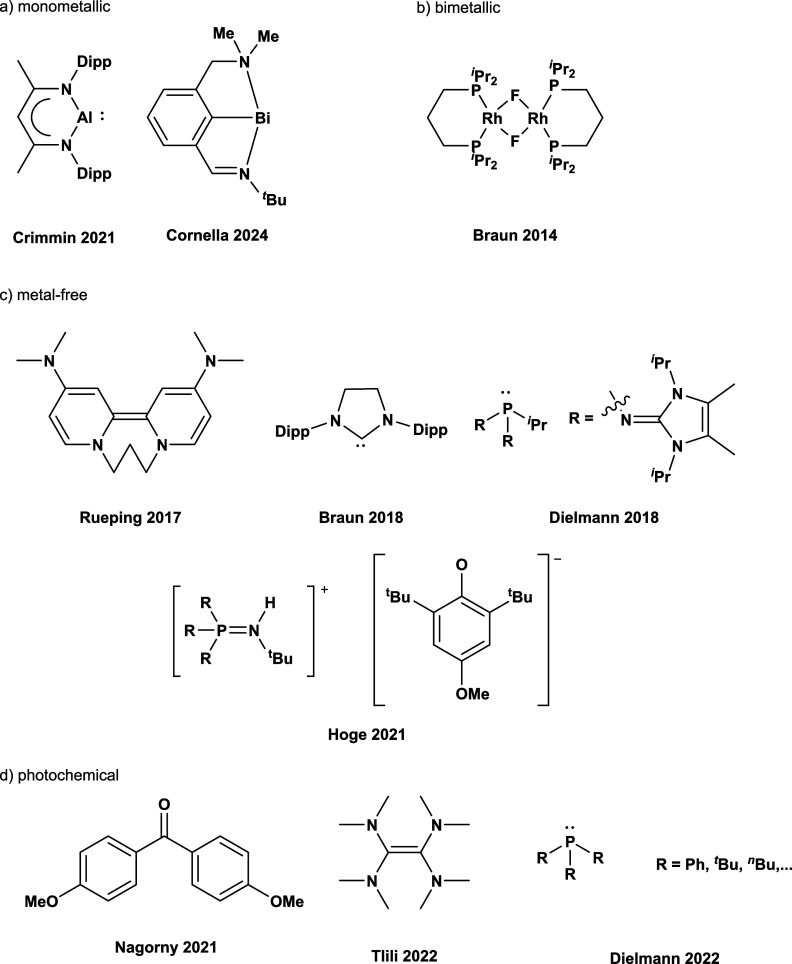
Selected compounds used in (a) monometallic,^14a,14c^ (b) bimetallic,^13a–13d^ (c) metal-free,^13e,13i^ and (d) photochemical^13e,13i^ approaches to activate SF_6_.

## Results and Discussion

In a J. Young NMR tube, a C_6_D_6_ solution of the bis­(gallylene) **1** ^16^ was allowed to react with an excess of SF_6_ at 80 °C, Scheme 1. Monitoring the reaction by ^1^H and ^19^F­{^1^H} NMR spectroscopy showed the full consumption of **1** after 8 h and the clean formation of a new compound featuring two singlets at −167.7 and −174.8 ppm in ^19^F­{^1^H} NMR spectrum as well as a distinct set of ^1^H resonances reminiscent of dinuclear bis­(β-diketiminate) gallium­(II) chloride and iodide complexes reported before.^17^ Notably, the reaction can also be performed at room temperature when a high intensity mercury arc lamp (maxima at 365, 405, and 436 nm, respectively; 140 W) is used, however, the reaction is less selective, Figures S9–S10. Despite the nominally symmetric geometry, the ^1^H NMR spectrum shows two distinct sets of resonances for the two β-diketiminate moieties due to fixed conformation of the *trans*-cyclohexylene-bridge; the γ-CH protons for example resonate as singlets at 4.54 and 4.61 ppm, respectively. Notably, other significant resonances did not appear neither in the ^1^H nor in the ^19^F­{^1^H} NMR spectrum and no precipitate could be observed. After a short workup, colorless crystals suitable for an X-ray diffraction analysis could be obtained from a concentrated *n*-hexane solution at −30 °C. The molecular structure of **2** in the solid state is shown in Figure 2 and resembles characteristics of the previously reported bis­(β-diketiminate) dihalodigallanes (Cl, I).^17^ The Ga–Ga and Ga–F bond lengths of 2.4115(6) Å and 1.8054(17) to 1.8204(18) Å, respectively, agree well with covalent gallium­(II)–gallium­(II) and gallium­(II)–fluorine bonds reported before.^16,17^ The F–Ga–Ga–F torsion angle of 22.1(1)°is in-line with the *syn*-periplanar conformation of the two fluorine substituents.

**Figure 2:**
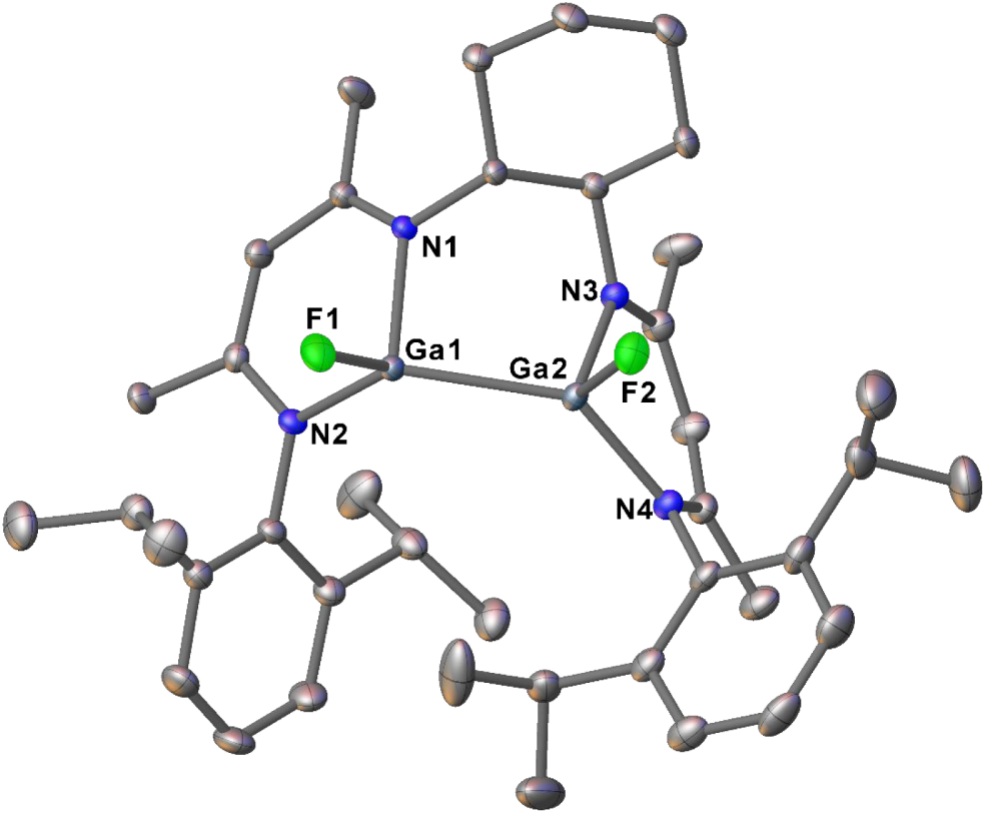
Solid-state molecular structure of **2** (ellipsoids represent 50% probability). Selected bond lengths (Å) and angles (°): Ga1–Ga2 2.4115(6), Ga1–F1 1.8204(18), Ga1–N1 1.988(2), Ga1–N2 1.939(2), Ga2–F2 1.8054(17), Ga2–N3 1.988(2), Ga2–N4 1.941(2), N1–Ga1–N2 97.34(9), N3–Ga2–N4 96.18(9). Hydrogen atoms are omitted for clarity. CCDC 2336539.

In analogy to the work by Crimmin and coworkers on the activation of SF_6_ by a monometallic allanediyl,^14a^ we considered that **1** might convert elemental sulfur into the respective sulfide. However, the reaction of **1** with S_8_ gives rise to a complex reaction mixture, most likely due to decomposition of **1**, Figure S11, and sulfide related signals could not been recognized in the crude reaction mixture of **1** and SF_6_. As the ^1^H and ^19^F­{^1^H} NMR spectra provide no indication where the sulfur remains, tri-*n*-butylphosphine (P­(^*n*^Bu)_3_) was added after the complete conversion of **1** aiming to trap elemental sulfur by oxidation to tri-*n*-butylphosphine sulfide (S=P­(^*n*^Bu)_3_). However, in the ^31^P­{^1^H} NMR spectrum only the signal of P­(^*n*^Bu)_3_ is present, so it is assumed that no sulfur was formed. Notably, in an NMR reaction of P­(^*n*^Bu)_3_ with elemental sulfur, tri-*n*-butylphosphine sulfide is formed selectively. When the reaction of **1** with SF_6_ was repeated in the presence of 1.0 eq. P­(^*n*^Bu)_3_, the formation of both S=P­(^*n*^Bu)_3_ and F_2_P­(^*n*^Bu)_3_ could be observed, although most of the P­(^*n*^Bu)_3_ remains unreacted. Hence, the intermediary formation of sulfur fluorides SF_*x*_ is likely to occur, which than react further with P­(^*n*^Bu)_3_. However, sulfur fluorides containing sulfur in a lower oxidation state such as SF_4_, SF_2_ or S_2_F_10_ could not be detected by ^19^F­{^1^H} NMR spectroscopy. Therefore, the reaction was repeated in the presence of one equivalent of 4-dimethylaminopyridine (4-DMAP), Figures S12–17, as it is known to form a stable 4-DMAP·SF_4_ adduct.^18^ After 30 min at room temperature, a color change from orange to red could be observed and monitoring by ^1^H and ^19^F­{^1^H} NMR spectroscopy showed the formation of **2**, which was completed after 16 h. Remarkably, 4-DMAP can also be used in catalytic/substochiometric, and the use of 0.1 equiv gave a complete conversion of **1** to **2** after 48 h, Figures S18–19. While this illustrates that 4-DMAP facilitates the activation of SF_6_ as reported before in case of the activation of C–F bonds,^19^ no resonances in account for a conceivable 4-DMAP·SF_4_ adduct^20^ were observed in the ^1^H or ^19^F­{^1^H} NMR spectra. In a direct reaction of **1** and 4-DMAP, we could not observe significant changes in the ^1^H NMR spectrum, Figure S20. Finally, we also conducted a trapping experiment with benzaldehyde but no formation of (difluoromethyl)­benzene, as expected if SF_4_ is formed, could be detected in the ^19^F NMR spectrum.

**Scheme 1:**
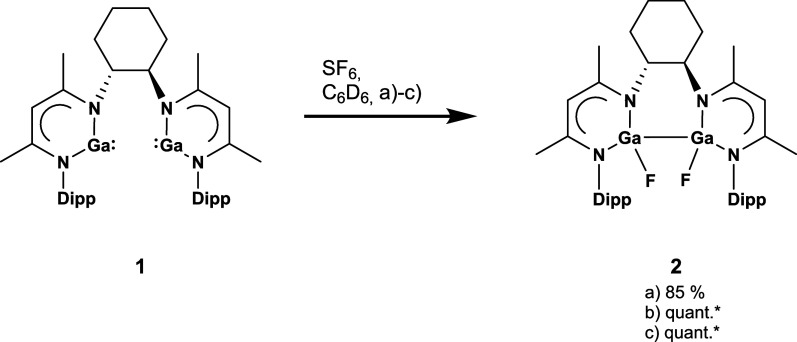
Synthesis of compound **2** under various conditions: (a) 80 °C, 8 h, (b) high intensity mercury arc lamp, 6 h, (c) 1 eq. 4-DMAP, r. t., 16 h; * = conversion determined by ^1^H NMR spectroscopy

Finally, we investigated whether the mononuclear gallium­(I) β-diketiminate **3** also reacts with SF_6_ or not, Scheme 2. However, and in agreement with our observations of the capabilities of **1** and **3** to activate aromatic C–F bonds,^16^ neither under the same thermal conditions nor in the presence of 4-DMAP, a reaction of **3** with SF_6_ was observed.

**Scheme 2:**
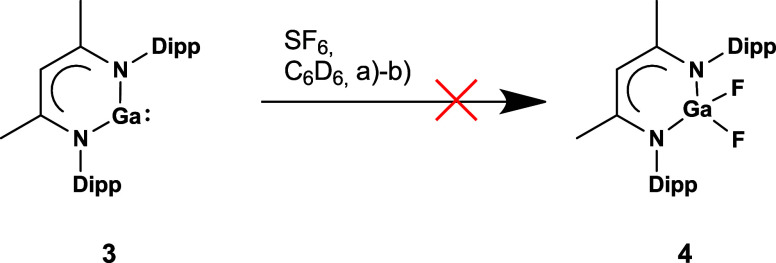
Attempted Synthesis of Compound **4** Under Various Conditions: (a) 80 °C, 16 h, (b) 1 eq. 4-DMAP, 80°C, 16 h

To gain further insight into the activation mechanism of SF_6_, a computational investigation was carried out at the DLPNO–CCSD­(T)/def2-TZVPP//M06–2X/def2-TZVP level of theory established previously.^16^ The computationally optimized structure of **2** shows good agreement with the corresponding molecular structure in the solid state as illustrated in Figure 2 (see Supporting Information, Table S3). As reported before,^16^ the highest occupied molecular orbital (HOMO, Figure 3B) is mainly composed of the σ-type donor orbitals at the Ga centers in an antibonding combination. Notably, the orientation of these MO lobes shows that the Ga^1^ (left) is directed toward Ga^2^ (right), suggesting electron donation from Ga^1^ to Ga^2^ (vide infra). This donor–acceptor interaction is further supported by the bonding combination of the orbitals as shown in the HOMO–1 (Figure 3A). Additionally, the LUMO+1 (Figure 3C) reveals a vacant p-type orbital centered on Ga^1^, highlighting its potential role as an acceptor site for further reactivity. The results obtained are consistent with previous work, differing primarily in the use of Natural Bond Orbitals instead of conventional frontier molecular orbitals as illustrated here.^16^

**Figure 3:**
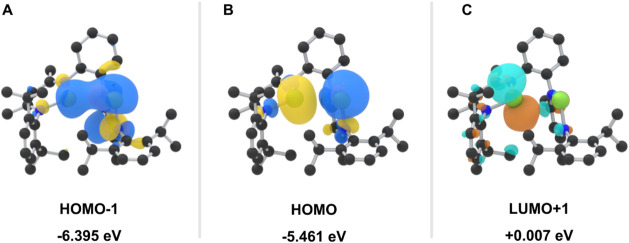
Relevant frontier molecular orbitals for the bis­(gallylene) **1**. The HOMO–1 (A) and HOMO (B) with isosurface values of 0.03, and 0.04 for the LUMO+1 (C).

To provide a quantitative extension of the molecular orbital analysis, Energy Decomposition Analysis (EDA)^21^ was performed to investigate the nature of the donor–acceptor interaction between Ga^1^ and Ga^2^. While **1** cannot directly be separated into fragments—as necessary for EDA—we created a model system by removing the linking cyclohexane and saturating the nitrogen atoms with hydrogen atoms. The resulting structure was reoptimized by relaxing only the hydrogens while keeping the core framework fixed. Moreover, the model system is justified, as full relaxation of the fragments reduces the Ga–Ga bond distance by only 0.067 Å, importantly without any repulsive elongation. Analysis of the EDA results reveals a bonding energy of −11.1 kJ·mol^–1^. The ratio of electrostatic (70%) and orbital (30%) contributions to the attractive energy terms is typical for a donor–acceptor bond (see Supporting Information, Table S3). This is supported by the analysis of the orbital relaxation term (Δ*E*_orb_) using Natural Orbitals for Chemical Valence (NOCV),^22^ which allows visualization of the deformation density and, in turn, the charge flow and nature of the bonding between the two Ga centers. The dominant energetic contribution between the two fragments represents the dative bond, which accounts for −15.3 kJ·mol^–1^ (31%) of the total Δ*E*_orb_. Further, examination of the charge flow of Δρ_1_ (Figure 4) reveals clear donation of electron density toward the Ga^2^ center from Ga^1^, specifically into the vacant delocalized π-orbitals of the N–Ga–N carbene-like moiety. These conclusions are further supported by the analysis of the Symmetrized Fragment Orbitals (SFOs) contributing to Δρ_1_ which quantitatively show the contributions from fragment orbitals to the deformation densities (see Supporting Information, Figure S27). The remaining energetic contributions consist of noncovalent interactions between the surrounding structure of the Ga centers, respectively.

**Figure 4:**
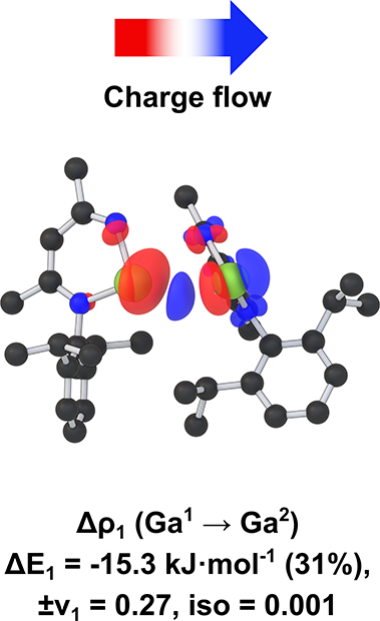
Most important deformation density contribution Δρ_1_ to the orbital relaxation term with energy (Δ*E*_1_) and eigenvalue (ν) in the EDA-NOCV analysis, illustrating the dative bonding interaction between the two Ga centers. Red denotes regions of electron density loss while blue represents regions of electron density gain.

Further calculations were performed utilizing the Nudged Elastic Band (NEB)^23^ approach to elucidate the mechanistic pathways with and without the presence of 4-DMAP. The results are presented as a schematic potential energy surface with Gibbs free energies in Figure 5A. First, we focused our investigations on the pathway (blue) starting with **1a**. The rate-limiting step of the mechanism is represented by **TS1**, which has a reaction barrier of 100.8 kJ·mol^–1^ relative to **1a**. Furthermore, **TS1** is considered an asymmetric concerted step in which the initial S–F^1^ bond activation occurs through the fluorination of the LUMO+1 acceptor site on Ga^1^ (Figure 3C). This electrophilic activation contrasts previous findings on the interplay between SF_6_ and Lewis-basic aluminum­(I)^14a^ or an electron-rich phosphane,^13b^ for which an S_N_2-type fluorine transfer has been computed as the initial step. The downhill of the minimum energy pathway (MEP) features the bond formation between the nucleophilic Ga^2^ center and the now electrophilic sulfur, leading to the formation of **Int1** and highlighting the cooperative nature of **1**. The final fluorination step to form **2a** occurs via a reductive elimination through **TS2**, which has the lowest computed free energy of activation in this pathway at +18.9 kJ·mol^–1^. Additionally, alternative fluorination positions were also evaluated, with corresponding transition states exhibiting barriers ranging from 19.3 to 30.9 kJ·mol^–1^ (see Supporting Information, Figure S33). This indicates that multiple positions are theoretically accessible for reductive elimination, with the most favorable site represented by **TS2** in Figure 5B. The slightly higher barriers observed at the other positions are likely due to a small increase in steric hindrance from the surrounding structure.

**Figure 5:**
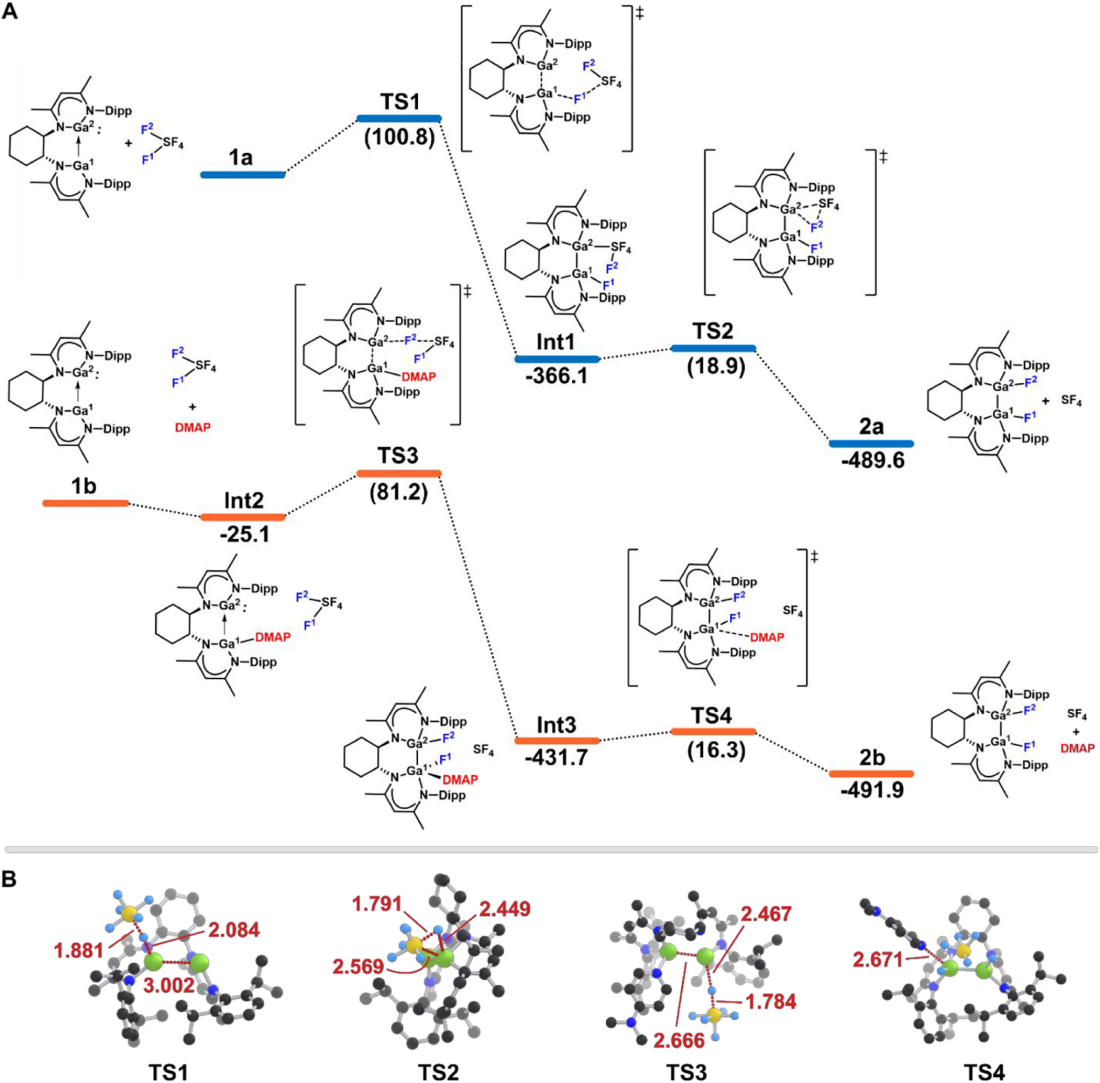
(A) Schematic potential-energy surface with Gibbs free energies at the DLPNO–CCSD­(T)/def2-TZVPP//M06–2X/def2-TZVP level of theory. The uncatalyzed pathway is represented in blue (top) while the 4-DMAP facilitated is in orange (bottom). All energies are given in kJ·mol^–1^. (B) Transition state structures illustrating the relevant bond distances in angstroms (Å).

We next focused on the 4-DMAP–facilitated pathway (orange), beginning with **1b**. Initial mechanistic investigations, calculated in a similar fashion as the unfacilitated pathway, considered 4-DMAP weakly coordinated to SF_6_ as found previously via a σ-hole interaction.^24^ However, this interaction proved insufficient to significantly weaken the S–F bonds, resulting in only a minimal reduction of the reaction barrier to 98.2 kJ·mol^–1^ and thus exceeding the threshold for reactivity under ambient conditions. Moreover, rather than coordinating to SF_6_, 4-DMAP was considered to bind to the LUMO+1 of the bis­(gallylene) **1**, leading to the formation of **Int2** in an exergonic process as previously reported for mononuclear gallylenes.^19c^ The corresponding TS could not be converged as the electronic barrier was exceptionally low (<15 kJ mol^–1^) in the resulting MEP from the NEB calculation (see Supporting Information, Figure S30). This is indicative that **Int2** forms spontaneously with no kinetic barrier. An examination of **Int2** reveals a decrease in the Ga–Ga bond distance compared to **1**, shortening from 3.263 Å to 2.950 Å. Further, this supports increased electron donation from Ga^1^ to Ga^2^, which enhances the nucleophilicity and reactivity of the Ga^2^ center. Indeed, when observing the activation energy of **TS3** it was found to decrease to 81.2 kJ·mol^–1^. As in the unfacilitated pathway, **TS3** proceeds via a concerted asymmetric transition state; however, in this case, activation occurs initially through the nucleophilic Ga^2^ center. The initial bond formation of Ga^2^–F^2^ arises first and then subsequently the Ga^1^–F^1^ bond formation occurs yielding **Int3**. This differs from the mechanism discussed above as fluorination occurs directly without the need for a reductive elimination step and thus producing SF_4_ after the initial rate-limiting step. To complete the mechanism, 4-DMAP dissociates from the Ga^1^ center through **TS4** with a small reaction barrier of +16.3 kJ·mol^–1^ to form the final complex **2b**. Ultimately, 4-DMAP is found to alter the activation mechanism of SF_6_ by binding to Ga^1^, which enhances electron donation towards Ga^2^ and thereby increases its nucleophilicity. This facilitates a direct asymmetric concerted fluorination of **Int2** via **TS3**, enabling the reaction to proceed at room temperature, in contrast to the unfacilitated pathway which requires elevated temperature, i.e., 80 °C.

To attempt investigating potential reaction byproducts, the rate-limiting transition states (**TS1** and **TS3**) and their preceding intermediates were reoptimized to elucidate the behavior of the intermediate sulfur species SF_4_ and SF_2_ (see Supporting Information, Section S3.5). Starting with the unfacilitated pathway through **TS1**, we observe a notable reduction in activation energy, with 82.4 kJ·mol^–1^ for SF_4_ and 55.0 kJ·mol^–1^ for SF_2_, respectively. A similar trend was observed for the 4-DMAP catalyzed pathway through **TS3**, where the free energy of activation is further decreased to 56.3 kJ·mol^–1^ for SF_4_ and 43.4 kJ·mol^–1^ for SF_2_. This suggests that the formation of elemental sulfur is theoretically accessible.

## Conclusions

In summary, we have investigated the reaction of the bis­(gallylene) **1** with sulfur hexafluoride under thermal and photochemical conditions. In either case, the formation of the bimetallic gallium­(II) fluoride **2** was observed, while the remaining of the sulfur could not be elucidated by NMR spectroscopy and trapping experiments. Using the Lewis base 4-dimethylaminopyridine (4-DMAP), the reaction already proceeds at room temperature. Computations shed light on the mechanistic pathways with and without the presence of 4-DMAP and provide a rational for the lower temperature required in the former case. The addition of 4-DMAP turns the electrophilic activation into a nucleophilic process and hence resembles a new general mode of bimetallic activation.

## Experimental Section

### General Information

All preparations were performed under an inert atmosphere of dinitrogen by means of standard Schlenk-line and glovebox (GS Systemtechnik and MBraun) techniques. Prior to usage, all glassware was stored in a 120 °C oven overnight. *n*-Pentane and *n*-hexane were distilled from LiAlH_4_. All solvents (toluene, *n*-hexane, C_6_D_6_, C_6_H_6_) were distilled from Na/benzophenone prior to use. **1** ^16^ and **3** ^25^ were synthesized according to literature procedures. Sodium (coated in a film of protective hydrocarbon oil, 99%) and benzophenone were purchased from Alfa Aesar. Sulfur hexaflouride 3.0 (99.9 Vol.% purity) was purchased from Westfalen Gas. 4-dimethylaminopyridine (99% purity) was purchased from TCI.

**Caution!** 4-Dimethylaminopyridine (CAS 1122–58–3) is acutely toxic (GHS05, 06, 08, and 09), and contact should be avoided using appropriate protective equipment or working in a glovebox.

### Characterization

The NMR spectra were recorded at room temperature (293 K) with a Bruker Avance III-500 MHz spectrometer or a Bruker Avance Neo-600 MHz spectrometer with δ referenced to external Si­(CH_3_)_4_ (^1^H and ^13^C­{^1^H}), CFCl_3_ (^19^F­{^1^H}) or H_3_PO_4_ (^31^P­{^1^H}) standard. ^1^H chemical shifts are referenced to the residual solvent signals of C_6_D_6_ (^1^H, 7.16 ppm). ^13^C chemical shifts are referenced to the residual solvent signals of C_6_D_6_ (^13^C, 128.06 ppm). The chemical shifts are reported in δ [ppm] and the coupling constants *J* are given in Hertz [Hz]. Assignment of the signals: s = singlet, d = doublet, q = quartet, sept = septet, m = multiplet.

Solution high-resolution mass spectrometry (HRMS) analysis was performed by Khrystyna Gerlach on a Bruker timsTOF MS System from Bruker Daltonik GmbH. The solid samples were prepared under an inert nitrogen atmosphere in a glovebox. The samples dissolved in dry dichloromethane were injected directly into the ion source at a flow rate of 180 μL/h. The samples were measured with an electrospray ionization (ESI) source in positive mode. An ESI-Tune Mix from Agilent was used for calibration; the calibration was performed in the mass range *m*/*z*: 50–3000.

ESI-source parameter: Scan Range *m*/*z*: 50–3000; Capillary Voltage: 4500 V; Nebulizer: 0.3 bar; Dry Gas Flow (N_2_): 3.0 L/min; Dry Heater Temperature: 180 °C. Quadrupole parameter for **2** are given in the Supporting Information.

IR spectra (given in cm^–1^) were recorded with an Agilent Cary 630 FT-IR spectrometer using a diamond ATR unit inside a nitrogen-filled glovebox.

### Synthesis

**2:** A J. Young NMR tube was charged with a solution of **1** (35 mg, 0.047 mmol) in C_6_D_6_ (0.4 mL). The solution was degassed once via freeze–pump–thaw and SF_6_ (0.5 bar) was added. The J. Young NMR tube was sealed and heated at 80 °C for 8 h. During this time the color changed from orange to yellow, and according to the ^1^H NMR spectra (Figures S1 and S6) **1** was completely converted. After the removal of C_6_D_6_ under *vacuo*, the remaining solid was washed with *n*-hexane (1 mL) and **2** could be obtained as a yellow solid (yield: 31 mg (85%)). Storing the *n*-hexane washings at −30 °C afforded colorless crystals suitable for an X-ray diffraction analysis.

^**1**^ **H NMR** (500 MHz, C_6_D_6_): δ [ppm] = 0.57 (d, ^3^ *J*_HH_ = 6.8 Hz, 3H, C*H*_3_ Dipp groups), 1.00 (d, ^3^ *J*_HH_ = 6.8 Hz, 3H, C*H*_3_ Dipp groups), 1.08 (d, ^3^ *J*_HH_ = 6.8 Hz, 3H, C*H*_3_ Dipp groups), 1.16 (d, ^3^ *J*_HH_ = 6.8 Hz, 3H, C*H*_3_ Dipp groups), 1.22 (d, ^3^ *J*_HH_ = 6.8 Hz, 3H, C*H*_3_ Dipp groups), 1.26 (d, ^3^ *J*_HH_ = 6.8 Hz, 3H, C*H*_3_ Dipp groups), 1.50 (d, ^3^ *J*_HH_ = 6.8 Hz, 3H, C*H*_3_ Dipp groups), 1.56 (s, 6H, CC*H*_3_), 1.57 (s, 3H, CC*H*_3_), 1.63 (d, ^3^ *J*_HH_ = 6.8 Hz, 3H, C*H*_3_ Dipp groups), 1.78 (s, 3H, CC*H*_3_), 2.15–2.07 (m, 1H, cyclohexyl C*H*_2_), 2.80 (sept, ^3^ *J*_HH_ = 6.8 Hz, 1H, C*H*(CH_3_)_2_ Dipp groups), 3.07 (sept, ^3^ *J*_HH_ = 6.8 Hz, 1H, C*H*(CH_3_)_2_ Dipp groups), 3.22 (sept, ^3^ *J*_HH_ = 6.8 Hz, 1H, C*H*(CH_3_)_2_ Dipp groups), 3.62–3.53 (m, 1H, cyclohexyl C*H*_2_), 3.92 (sept, ^3^ *J*_HH_ = 6.8 Hz, 1H, C*H*(CH_3_)_2_ Dipp groups), 4.54 (s, 1H, methine C*H*), 4.61 (s, 1H, methine C*H*), 4.84–4.74 (m, 1H, cyclohexyl C*H*_2_), 6.91 (d, ^3^ *J*_HH_ = 7.4 Hz, 1H, aromatic proton of Dipp groups), 7.23–7.07 (m, aromatic protons of Dipp groups and residual solvent peaks).

^**13**^ **C­{** ^**1**^ **H} NMR** (126 MHz, C_6_D_6_): δ [ppm] = 21.3 (C*C*H_3_), 23.1 (*C*H_3_ Dipp groups), 23.6 (C*C*H_3_), 24.0 (*C*H_3_ Dipp groups), 24.2 (C*C*H_3_), 24.6 (*C*H_3_ Dipp groups), 24.8 (*C*H_3_ Dipp groups), 25.1 (*C*H_3_ Dipp groups), 25.3 (*C*H_3_ Dipp groups), 25.7 (*C*H_3_ Dipp groups), 25.8 (*C*H_3_ Dipp groups), 27.8 (*C*H­(CH_3_)_2_ Dipp groups), 28.5 (*C*H­(CH_3_)_2_ Dipp groups), 29.4 (*C*H­(CH_3_)_2_ Dipp groups), 29.6 (*C*H­(CH_3_)_2_ Dipp groups), 32.0 (cyclohexyl *C*H_2_), 32.6 (cyclohexyl *C*H_2_), 34.1 (cyclohexyl *C*H_2_), 35.0 (cyclohexyl *C*H_2_), 62.4 (cyclohexyl *C*H), 62.5 (cyclohexyl *C*H), 96.7 (methine *C*H), 98.4 (methine *C*H), 123.9, 124.4, 124.5, 125.6, 127.1, 127.3, 128.0, 128.2, 139.5 (*C* Dipp groups), 142.2 (*C* Dipp groups), 142.6 (*C* Dipp groups), 143.8 (*C* Dipp groups), 145.4 (*C* Dipp groups), 147.2 (*C* Dipp groups), 164.9 (*C*CH_3_), 166.1 (*C*CH_3_), 166.2 (*C*CH_3_), 167.8 (*C*CH_3_).

^**19**^ **F­{** ^**1**^ **H} NMR** (565 MHz, C_6_D_6_): δ [ppm] = −167.7 (Ga*F*), −174.8 (Ga*F*).

**IR** ν [cm^–1^] = 3058, 2959, 2923, 2864, 1560, 1511, 1457, 1435, 1380, 1319, 1263, 1191, 1131, 1094, 1055, 1019, 935, 867, 798, 759, 633, 575, 537, 499.

**HRMS** (ESI +): *m*/*z* calculated for [C_40_H_58_Ga_2_F_2_N_4_]-F^+^: 753.3151 (M-F*^+^); *m*/*z* found: 753.3152 (M-F*^+^).

#### Alternative Synthetic Routes Toward 2

a) A J. Young NMR tube was charged with a solution of **1** (30 mg, 0.041 mmol) in C_6_D_6_ (0.4 mL). The solution was degassed once via freeze–pump–thaw and SF_6_ (0.5 bar) was added. The sample was irradiated with a high intensity mercury arc lamp for 6 h. During this time the color changed from orange to yellow, and according to the ^1^H NMR spectrum (Figure S9) **1** was completely converted.
b) A J. Young NMR tube was charged with a solution of **1** (30 mg, 0.041 mmol) and 5 mg (0.041 mmol, 1.0 equiv) of 4-DMAP in C_6_D_6_ (0.4 mL). The solution was degassed once via freeze–pump–thaw and SF_6_ (0.5 bar) was added. The orange solution was mixed with a vertical NMR carousel rotating shaker at room temperature. After 30 min the color changed to red and after the reaction was finished the color was yellow, according to the ^1^H NMR spectrum (Figure S12) **1** was completely converted after 16 h.
c) A J. Young NMR tube was charged with a solution of **1** (30 mg, 0.041 mmol) and 5 mg (0.004 mmol, 0.1 equiv) of 4-DMAP in C_6_D_6_ (0.4 mL). The solution was degassed once via freeze–pump–thaw and SF_6_ (0.5 bar) was added. The orange solution was mixed with a vertical NMR carousel rotating shaker at room temperature. During this time the color changed from orange to yellow, and according to the ^1^H NMR spectrum (Figure S18) **1** was completely converted after 48 h.

#### Synthesis of **2** with One Equivalent of SF_6_

a) A J. Young NMR tube was charged with a solution of **1** (15 mg, 0.020 mmol, 1.0 equiv) in C_6_D_6_ (0.4 mL) and SF_6_ (0.46 mL, 0.020 mmol, 1.0 equiv) was added with a syringe. The J. Young NMR tube was heated at 80 °C for 25.5 h. During this time the color changed to a slightly deeper orange, but only small amounts of **2** could be observed according to the ^1^H NMR spectrum (Figure S14).
b) A J. Young NMR tube was charged with a solution of **1** (15 mg, 0.002 mmol, 1.0 equiv) and 2.5 mg (0.020 mmol, 1.0 equiv) of 4-DMAP in C_6_D_6_ (0.4 mL). SF_6_ (0.46 mL, 0.020 mmol, 1.0 equiv) was added with a syringe and the orange solution was mixed with a vertical NMR carousel rotating shaker at room temperature for 24 h. After 1 h the color changed to red and after the reaction was finished the color was yellow, according to the ^1^H NMR spectrum (Figure S16) **1** was completely converted.

#### Reactions with P­(^*n*^Bu)_3_ for the Determination of Byproducts

a) A J. Young NMR tube was charged with a solution of **1** (20 mg, 0.027 mmol, 1.0 equiv) in C_6_D_6_ (0.4 mL). The solution was degassed once via freeze–pump–thaw and SF_6_ (0.5 bar) was added. The J. Young NMR tube was sealed and heated at 80 °C for 9 h. During this time the color changed from orange to yellow, and according to the ^1^H NMR spectrum, **1** was completely converted. To the reaction solution P­(^*n*^Bu)_3_ (6.7 μL, 0.027 mmol, 1.0 equiv) was added and the solution was mixed with a vertical NMR carousel rotating shaker at room temperature for 2 h. According to the ^31^P­{^1^H} NMR spectrum (Figure S21) no S = P­(^*n*^Bu)_3_ was formed.
b) A J. Young NMR tube was charged with a solution of **1** (30 mg, 0.027 mmol, 1.0 equiv) and P­(^*n*^Bu)_3_ (10.1 μL, 0.041 mmol, 1.0 equiv) in C_6_D_6_ (0.4 mL). The solution was degassed once via freeze–pump–thaw and SF_6_ (0.5 bar) was added. The J. Young NMR tube was sealed and heated at 80 °C for 17 h and according to the ^1^H NMR spectrum (Figure S22) **1** was completely converted. According to the ^31^P­{^1^H} NMR spectrum (Figure S23) most of P­(^*n*^Bu)_3_ remained unreacted and only a small amount of S=P­(^*n*^Bu)_3_ and some traces of difluoro-tri-*n*-butylphosphine (F_2_P­(^*n*^Bu)_3_) were formed.

#### Reaction of **1** with Elemental Sulfur

A J. Young NMR tube was charged with a solution of **1** (20 mg, 0.027 mmol) and 1.8 mg (0.007 mmol, 0.25 equiv) of S_8_ in C_6_D_6_ (0.4 mL). The orange solution was mixed with a vertical NMR carousel rotating shaker at room temperature for 16 h. According to the ^1^H NMR spectrum (Figure S11) **1** was completely decomposed.

#### Reaction of **1** with One Equivalent of 4-DMAP

A J. Young NMR tube was charged with a solution of **1** (30 mg, 0.041 mmol) and 5 mg (0.041 mmol, 1.0 equiv) of 4-DMAP in C_6_D_6_ (0.4 mL). The orange solution was mixed with a vertical NMR carousel rotating shaker at room temperature for 16 h, but no conversion of **1** could be observed according to the ^1^H NMR spectrum (Figure S20).

#### Reactions with Benzaldehyde

A pressure tube was charged with a solution of **1** (35 mg, 0.048 mmol, 1.0 equiv) in C_6_H_6_ (2 mL). The solution was degassed once via freeze–pump–thaw and SF_6_ (0.5 bar) was added. The pressure tube was sealed and heated at 80 °C for 16 h. The solution was frozen in liquid nitrogen and benzaldehyde (5.1 mg, 0.048 mmol, 1.0 equiv) was added. After warming up to room temperature, the solution was heated at 80 °C for 10 min. For recording an NMR, 0.2 mL of the reaction solution was taken and diluted with 0.2 mL of C_6_D_6_. According to the ^19^F NMR spectrum (Figure S25) no (difluoromethyl)­benzene was formed as expected for the reaction of possibly formed SF_4_ with benzaldehyde.

#### Reactions of **3** with SF_6_

a) A J. Young NMR tube was charged with a solution of **3** (10 mg, 0.021 mmol) in C_6_D_6_ (0.4 mL). The solution was degassed once via freeze–pump–thaw and SF_6_ (0.5 bar) was added. The J. Young NMR tube was sealed and heated at 80 °C for 16 h, but no conversion of **3** could be observed according to the ^1^H NMR spectrum (Figure S26).
b) A J. Young NMR tube was charged with a solution of **3** (10 mg, 0.021 mmol, 1.0 equiv) and 4-DMAP (2.6 mg, 0.021 mmol, 1.0 equiv) in C_6_D_6_ (0.4 mL). The solution was degassed once via freeze–pump–thaw and SF_6_ (0.5 bar) was added. The J. Young NMR tube was sealed and heated at 80 °C for 16 h, but no conversion of **3** could be observed according to the ^1^H NMR spectrum (Figure S28).

## Computational Details

The initial starting structure of bis­(gallylene) **1** used in this work was taken from previous computational studies.^16^ Manipulation of this structure to create various intermediates and final structures was done using the Avogadro 2 (version 1.101.0) graphical user interface.^26^ All geometry optimizations were carried out using ORCA 6.0.0 ^27^ employing the M06–2X density functional^28^ along with the def2-TZVP basis set^29^ as established in a previous study.^16^ All the calculations used a convergence criterion for the self-consistent field (SCF) cycles of 10^–8^ Hartree (*E*_h_) change in energy. The convergence tolerances for the geometry optimizations were set as follows: the energy change threshold was 5 × 10^–6^ *E*_h_, the gradient criteria included a root-mean-square (RMS) value of 10^–4^ *E*_h_·bohr^–1^ and a maximum gradient threshold of 3 × 10^–4^ *E*_h_·bohr^–1^, while the step sizes were constrained with an RMS step tolerance of 2 × 10^–3^ bohr and a maximum tolerance of 4 × 10^–3^ bohr. Additionally, the default grid settings (DefGrid2) were used for the geometry optimizations. Frequency calculations were performed to ensure that all optimized structures correspond to true minima on the potential energy surface, as indicated by the absence of imaginary frequencies. If imaginary modes were present, a finer integration grid (DefGrid3) was applied and the optimization thresholds were tightened: the energy change criterion was set to 10^–6^ *E*_h_, gradients were required to meet an RMS of 3 × 10^–5^ *E*_h_·bohr^–1^ and a maximum of 10^–4^ *E*_h_·bohr^–1^, while step sizes were limited to an RMS of 6 × 10^–4^ bohr and a maximum of 10^–3^ bohr. All the resulting structures underwent a final frequency calculation at both 298.15 and 353.15 K to give the required Gibbs free energy corrections needed for higher level single point calculations via statistical thermodynamics approach using the double harmonic approximation (vida infra).

Using the optimized reactants, intermediates, and products, the nudged elastic band (NEB)^23a^ approach was employed to elucidate the transition states (TS) for the two reaction mechanisms investigated. Initial images along the minimum energy pathway (MEP) were generated using the Geodesic Interpolation Python code,^30^ providing an improved starting point for the MEP. Climbing image (CI) NEB calculations^23b^ were carried out using the Limited-memory Broyden–Fletcher–Goldfarb–Shanno (LBFGS)^31^ optimization algorithm with a spring constant between the images of the MEP of 10^–2^ *E*_h_·bohr^–2^. Prior to switching to the CI-NEB calculation, the NEB was allowed to minimize until the maximum perpendicular force of the images to the MEP decreased below the threshold of 2 × 10^–2^ *E*_h_·bohr^–1^. The highest energy image was then designated as the CI, and the CI-NEB calculation was considered converged when the RMS of the perpendicular forces across all images was beneath the threshold of 2.5 × 10^–3^ *E*_h_·bohr^–1^ with a maximum of 5 × 10^–3^ *E*_h_·bohr^–1^. Moreover, the force on the CI is further restricted to an RMS of 2.5× 10^–4^ *E*_h_·bohr^–1^ and a maximum of 5 × 10^–4^ *E*_h_·bohr^–1^.

After achieving convergence in the CI-NEB calculation, the CI structure was further refined through a TS optimization at the same level of theory. An eigenvector-following algorithm with a P-RFO step was employed,^32^ tracking the smallest vibrational mode (i.e., the largest imaginary frequency) from the CI in the uphill direction while allowing the remaining geometry to relax. Additionally, the same standard SCF and optimization convergence criteria, as mentioned prior, were applied to the TS optimizations. Following relaxation of the TS geometry, a subsequent frequency calculation was performed to verify the presence of a single imaginary mode, confirming that a transition state corresponding to a first-order saddle point on the potential energy surface had been obtained.

Once the geometries of all reactants, intermediates, TSs, and products were obtained, the domain-based local pair natural orbital coupled cluster method [DLPNO–CCSD­(T)][Bibr ref33] was applied for higher-level single-point energy calculations. The def2-TZVPP basis set employed along with its respective auxiliary basis sets.^34^ The SCF convergence threshold for these calculations were increased to an energy change of 10^–6^ *E*_h_. Furthermore, the previously obtained Gibbs free energy corrections based on DFT computations were added to the electronic energies derived from the latter DLPNO–CCSD­(T) calculations. Ultimately, this provided all the Gibbs free energies required to construct the schematic potential-energy surface.

To gain further insight on the dative nature of the Ga–Ga bond, energy decomposition (EDA) was employed using the AMS 2024.102 ^35^ software package. Prior to the EDA, a model system was created by removing the linking cyclohexane and saturating the nitrogen atoms with hydrogen. A geometry optimization only relaxing the positions of the hydrogen atoms using ORCA was performed on the model system using the same standard thresholds described earlier to ensure that the saturating hydrogen atoms occupied reasonable positions. The EDA calculation was then performed using the scheme developed by Morokuma^21a^ as well as Ziegler and Rauk^21b,21c^ (see Supporting Information Section S3.2 for definitions of bonding energy components). Here, the fragments were defined as the now two separate Ga structures. The same M06-2X functional was applied with the TZP Slater-type orbital basis set.^36^ No frozen core approximations were used, and ZORA scalar relativistic effects^37^ were applied along with “Normal” numerical quality calculation parameters. Natural Orbitals for Chemical Valence (NOCV)^22^ were enabled in all EDA calculations to visualize the orbital interactions between the two fragments. The minimum energy threshold for the NOCV pairs was set to 2.0 kcal·mol^–1^, with an eigenvalue threshold of 0.05. Additionally, the population analysis threshold for individual symmetry fragment orbital contributions to each deformation density was set to 0.01.

## Supplementary Material



## Notes

The following files are available free of charge: The Supporting Information is available free of charge at https://pubs.acs.org/doi/10.1021/acs.inorgchem.5c04924.

Experimental, crystallographic and computational details as well as NMR and IR spectra (PDF)

## Abbreviations

4-DMAP: 4-dimethylaminopyridine
^*n*^Bu: *n*-butyl
Dipp: 2,6-diisopropylphenyl
Me: methyl
^*t*^Bu: *tert*-butyl
^*i*^Pr: *iso*-propyl
IR: infrared
HRMS: high resolution mass spectrometry
*m*/*z*: mass-to-charge
NMR: nuclear magnetic resonance
sc-XRD: single-crystal X-ray diffraction.

## References

[1] Christophorou L. G., Olthoff J. K., van Brunt R. J. (1997). Sulfur hexafluoride and the electric
power industry. IEEE Electr. Insul. Mag..

[2] Maiss M., Brenninkmeijer C. A.
M. (1998). Atmospheric SF_6_: Trends, Sources, and Prospects. Environ.
Sci. Technol..

[3] Bouvet S., Pégot B., Sengmany S., Le Gall E., Léonel E., Goncalves A.-M., Magnier E. (2020). Controlled decomposition of SF_6_ by electrochemical reduction. Beilstein
J. Org. Chem..

[4] Rombach D., Wagenknecht H.-A. (2018). Photoredox Catalytic Activation of
Sulfur Hexafluoride for Pentafluorosulfanylation of α-Methyl-
and α-Phenyl Styrene. ChemCatchem.

[5] Cowen H. C., Riding F., Warhurst E., Buckingham A. D., Le Fèvre R. J.
W., Meakins G. D., Haszeldine R. N., Jander J., Clemo G. R., Fox B. W., Raper R., McOmie J. F. W., White I. M., Brady O. L., Halstead P. E., Sharpe A. G., Gray G. W., Jones B., Cast J., Stevens T. S., Bell F., Cook J. W., Hunter L., Barrer R. M., Mackenzie N., MacLeod D., Barker C. C., Casson F. D., Laws G. F., Carruthers W., Lane E. S., Williams C. (1953). The Reaction of Sulphur
Hexafluoride
with Sodium. J. Chem. Soc..

[6] Dirican D., Pfister N., Wozniak M., Braun T. (2020). Reactivity of Binary
and Ternary Sulfur Halides towards Transition-Metal Compounds. Chem. Eur. J..

[7] Harvey B. G., Arif A. M., Glöckner A., Ernst R. D. (2007). SF_6_ as
a Selective and Reactive Fluorinating Agent for Low-Valent Transition
Metal Complexes. Organometallics.

[8] McTeague T. A., Jamison T. F. (2016). Photoredox Activation
of SF_6_ for Fluorination. Angew. Chem.,
Int. Ed..

[9] Holze P., Horn B., Limberg C., Matlachowski C., Mebs S. (2014). The activation of sulfur hexafluoride at highly reduced low-coordinate
nickel dinitrogen complexes. Angew. Chem., Int.
Ed..

[10] Berg C., Braun T., Ahrens M., Wittwer P., Herrmann R. (2017). Activation
of SF_6_ at Platinum Complexes: Formation of SF_3_ Derivatives and Their Application in Deoxyfluorination Reactions. Angew. Chem., Int. Ed..

[11] Zámostná L., Braun T., Braun B. (2014). S-F and S-C activation of SF_6_ and SF_5_ derivatives
at rhodium: Conversion of SF_6_ into H_2_S. Angew. Chem., Int. Ed..

[12] Basta R., Harvey B. G., Arif A. M., Ernst R. D. (2005). Reactions
of SF_6_ with organotitanium and organozirconium complexes:
The “inert”
SF_6_ as a reactive fluorinating agent. J. Am. Chem. Soc..

[13] Rueping M., Nikolaienko P., Lebedev Y., Adams A. (2017). Metal-free
reduction of the greenhouse
gas sulfur hexafluoride, formation of SF_5_ containing ion
pairs and the application in fluorinations. Green Chem..

[14] Sheldon D. J., Crimmin M. R. (2021). Complete Deconstruction
of SF_6_ by an Aluminium­(I) Compound. Chem. Commun..

[15] Deubner H., Kraus F. (2017). The Decomposition Products of Sulfur Hexafluoride (SF_6_) with Metals Dissolved in Liquid Ammonia. Inorganics.

[16] Kysliak O., Görls H., Kretschmer R. (2021). Cooperative Bond Activation by a
Bimetallic Main-Group Complex. J. Am. Chem.
Soc..

[17] Helling C., Döhler L., Kysliak O., Görls H., Liebing P., Wölper C., Kretschmer R., Schulz S. (2024). Metal-metal cooperativity boosts Lewis basicity and
reduction properties of the bis­(gallanediyl) ^Cy^L_2_Ga_2_. Dalton Trans..

[18] Chaudhary P., Goettel J. T., Mercier H. P. A., Sowlati-Hashjin S., Hazendonk P., Gerken M. (2015). Lewis acid behavior of SF_4_: Synthesis, characterization,
and computational study of adducts
of SF_4_ with pyridine and pyridine derivatives. Chem. Eur. J..

[19] Lv H., Zhan J.-H., Cai Y.-B., Yu Y., Wang B., Zhang J.-L. (2012). π-π interaction assisted
hydrodefluorination of perfluoroarenes by gold hydride: A case of
synergistic effect on C-F bond activation. J.
Am. Chem. Soc..

[20] Pavone M., Barone V., Ciofini I., Adamo C. (2004). First-principle molecular
dynamics of the Berry pseudorotation: Insights on ^19^F NMR
in SF_4_. J. Chem. Phys..

[21] Kitaura K., Morokuma K. (1976). A new energy decomposition
scheme for molecular interactions within the Hartree-Fock approximation. Int. J. Quantum Chem..

[22] Mitoraj M., Michalak A. (2007). Natural orbitals for
chemical valence as descriptors of chemical bonding in transition
metal complexes. J. Mol. Model..

[23] a Jónsson, H. ; Mills, G. ; Jacobsen, K. W. Nudged elastic band method for finding minimum energy paths of transitions. In Classical and Quantum Dynamics in Condensed Phase Simulations. Berne, B. J. ; Ciccotti, G. ; Coker, D. F. , Eds.; World Scientific, 1998; pp. 385–404. 10.1142/9789812839664_0016.

[24] Bittner D. M., Zaleski D. P., Stephens S. L., Walker N. R., Legon A. C. (2015). The σ-hole
interaction between sulfur hexafluoride and ammonia characterised
by broadband rotational spectroscopy. ChemPhyschem.

[25] Kysliak O., Görls H., Kretschmer R. (2020). Salt metathesis as an alternative
approach to access aluminium­(I) and gallium­(I) β-diketiminates. Dalton Trans..

[26] Kitware Open Chemistry. https://www.openchemistry.org/.

[27] Neese F. (2012). The ORCA program system. WIREs Comput. Mol. Sci..

[28] Zhao Y., Truhlar D. G. (2008). The M06 suite of
density functionals for main group
thermochemistry, thermochemical kinetics, noncovalent interactions,
excited states, and transition elements: Two new functionals and systematic
testing of four M06-class functionals and 12 other functionals. Theor. Chem. Acc..

[29] Weigend F., Ahlrichs R. (2005). Balanced basis sets of split valence,
triple zeta valence
and quadruple zeta valence quality for H to Rn: Design and assessment
of accuracy. Phys. Chem. Chem. Phys.

[30] Zhu X., Thompson K. C., Martínez T. J. (2019). Geodesic
interpolation for reaction
pathways. J. Chem. Phys..

[31] Liu D. C., Nocedal J. (1989). On the limited memory
BFGS method for large scale optimization. Math.
Program..

[32] Baker J. (1986). An algorithm
for the location of transition states. J. Comput.
Chem..

[33] Riplinger C., Neese F. (2013). An efficient and near linear scaling pair natural orbital based local
coupled cluster method. J. Chem. Phys..

[34] Weigend F., Köhn A., Hättig C. (2002). Efficient use of the correlation
consistent basis sets in resolution of the identity MP2 calculations. J. Chem. Phys..

[35] Software for Chemistry and Materials Accelerate your chemistry and materials research. http://www.scm.com/.

[36] van Lenthe E., Baerends E. J. (2003). Optimized Slater-type
basis sets for the elements 1–118. J.
Comput. Chem..

[37] van
Lenthe E., van Leeuwen R., Baerends E. J., Snijders J. G. (1996). Relativistic
regular two-component Hamiltonians. Int. J.
Quantum Chem..

