# Supplementary material for: Bimetallic Activation of SF6 by a Bis(Gallylene)
Source: Inorg Chem. 2025 Dec 8;64(50):24948–57. doi: 10.1021/acs.inorgchem.5c04924 (PMC12728938; doi:10.1021/acs.inorgchem.5c04924)
Supplement: Supplementary file 1 [file ic5c04924_si_001.pdf]

Bimetallic activation of SF_6 by a bis(gallylene)

Aylin Nagel,[a] Max Neubauer,[a] Douglas L. Miller,[b] Phil Köhler,[c] Ralf Tonner-Zech,[b] and Robert Kretschmer*[a,d]*

a) A. Nagel, M. Neubauer, Prof. Dr. R. Kretschmer, Institut für Chemie, Technische Universität Chemnitz, Strasse der Nationen 62, 09111 Chemnitz, Germany

b) D. L. Miller, Prof. Dr. R. Tonner-Zech, Wilhelm-Ostwald-Institut für Physikalische und Theoretische Chemie, Universität Leipzig, Linnéstr. 2, 04103 Leipzig, Germany, E-Mail: ralf.tonner@uni-leipzig.de

c) Dr. P. Köhler, Institut für anorganische und analytische Chemie, Friedrich-Schiller-Universität Jena, Humboldtstraße 8, 07443 Jena, Germany

d) Prof. Dr. R. Kretschmer, Jena Center of Soft Matter, Friedrich-Schiller-Universität Jena, Philosophenweg 7, 07443 Jena, Germany, E-mail: robert.kretschmer@chemie.tu-chemnitz.de

Table of contents

1. Experimental and crystallographic details 3

1.1. Mass spectrometry 3

1.2. Crystallographic Details 4

2. NMR and IR Spectra 6

3. Computational Details 21

3.1. Bonding Parameters Comparison to XRD Data 21

3.2. Energy Decomposition Analysis Data 21

3.3. Nudged Elastic Band Minimum Energy Pathways 23

3.4. Reductive Elimination Site Investigation 26

3.5. Activation Barriers of SF_x Intermediates 27

4. References 28

1. Experimental and crystallographic details

1.1. Mass spectrometry

Table S1. Quadrupole Parameter for compound **2**.

	**2**
Ion Energy [eV]	5.0
Collision Energy [eV]	2.0
Collision Cell RF [Vpp]	1000.0
Transfer Time [µs]	80.0
PrePulse Storage Time [µs]	10.0

1.2. Crystallographic Details

The single-crystal X-ray intensity data of **2** were collected on a Bruker-Nonius Kappa-CCD diffractometer using Mo-K_{α} radiation (λ = 0.71073 Å), at 120(2) K. Multi-scan absorption correction was applied to the intensity data.[S1] The structure was solved with SHELXT-2018/3[S2] and refined by full matrix least-squares methods on F^2 with SHELXL-2018/3[S3], using the Olex2 1.3 environment[S4]. The crystal was treated as an inversion twin.

CCDC 2336539 contain supplementary crystallographic data for **2**. These data can be obtained free of charge from The Cambridge Crystallographic Data Centre via www.ccdc.cam.ac.uk.

Table S2. Crystal and structural refinement data of **2**.

	**2**
Empirical formula	$C_{40}H_{58}F_2Ga_2N_4$
Formula mass [g·mol^{-1}]	772.34
Crystal system	Orthorhombic
Space group	$P2_12_12_1$
a [Å]	8.942(2)
b [Å]	11.456(3)
c [Å]	38.502(9)
α [°]	90
β [°]	90
γ [°]	90
V [Å^3]	3944.1(16)
Z	4
ρ_{calc} [g·cm^{-3}]	1.301
Absorption coefficient [mm^{-1}]	1.407
F(000)	1624.0
Reflection collected	48201
Reflection unique / R_{int} [a)]	11802 / 0.0358
Goodness of fit on F^2 [b)]	1.069
Data / restrains / parameters	11802 / 0 / 446
Θ range for data collection [°]	3.71 to 62.616
Limiting indices	$-13 \leq h \leq 13$,
	$-16 \leq k \leq 16$,
	$-54 \leq l \leq 52$
Final R indices [$I > 2\sigma(I)$][c)]	R_1 = 0.0316, wR_2 = 0.0715
R indices (all data)[c)]	R_1 = 0.0352, wR_2 = 0.0726
Largest diff. peak/hole [e·Å^3]	0.43/–0.57

[a)] $R_{int} = \sum|F_o^2 - F_o^2(mean)| / \sum F_0^2$ where F_o^2 (mean) is the average intensity of symmetry equivalent diffractions. [b)] $S = \left[\sum w\left(F_o^2 - F_c^2\right)^2 / N_{\text{Ref.}} - N_{\text{Par.}}\right]^{1/2}$, where $N_{\text{Ref.}}$ = number of independent reflections, $N_{\text{Par.}}$ = number of parameters. [c)] $R_1 = \left[\sum||F_o| - |F_c||\right] / \sum|F_o|$; $wR_2 = \left[\left(\sum w\left(F_o^2 - F_c^2\right)^2\right) / \sum w\left(F_o^2\right)^2\right]^{1/2}$

2. NMR and IR Spectra

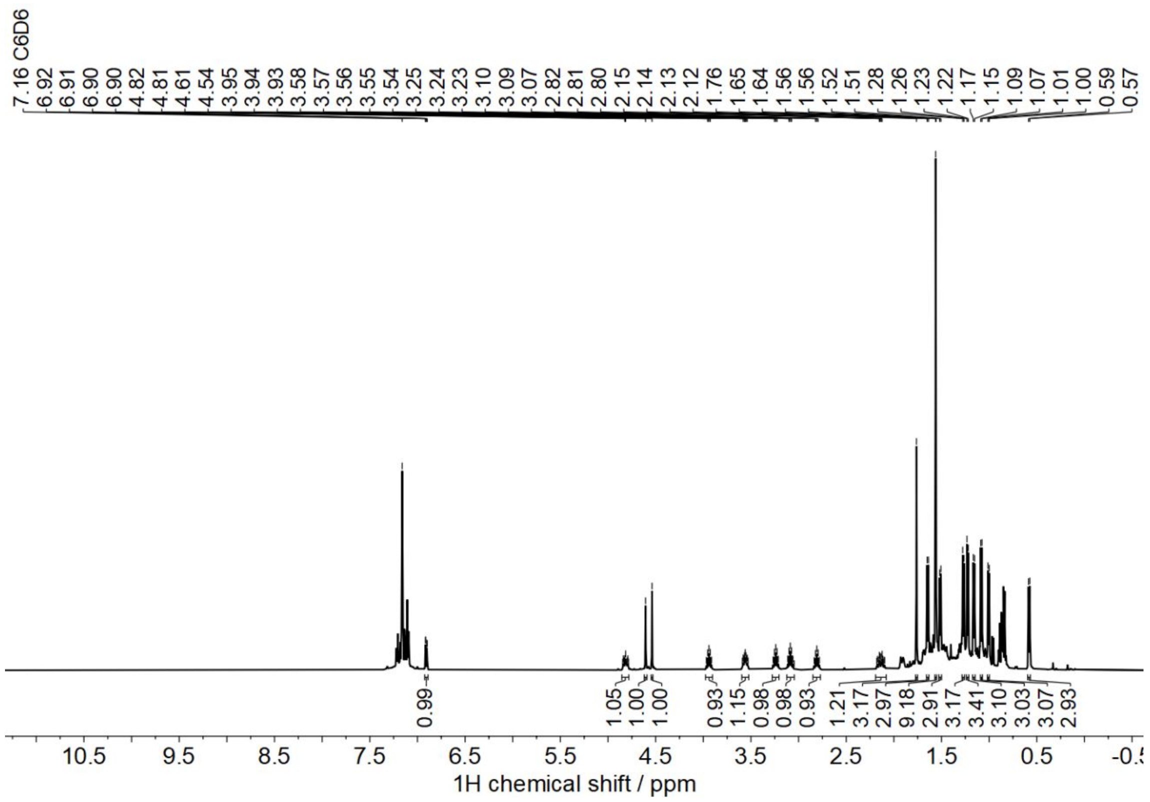

Figure S1. ^{1}H NMR spectrum (500 MHz, C_6D_6, 293 K) of **2**.

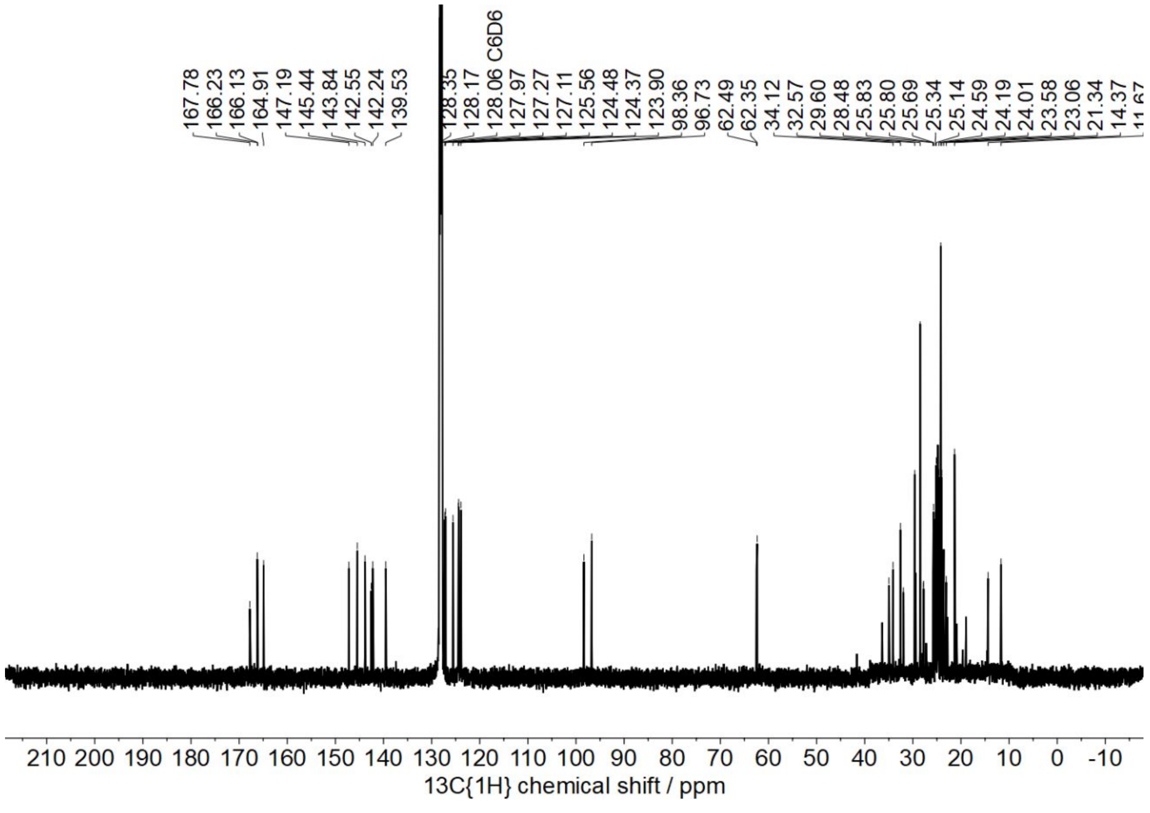

Figure S2. $^{13}C\{^{1}H\}$ NMR spectrum (126 MHz, C_6D_6, 293 K) of **2**.

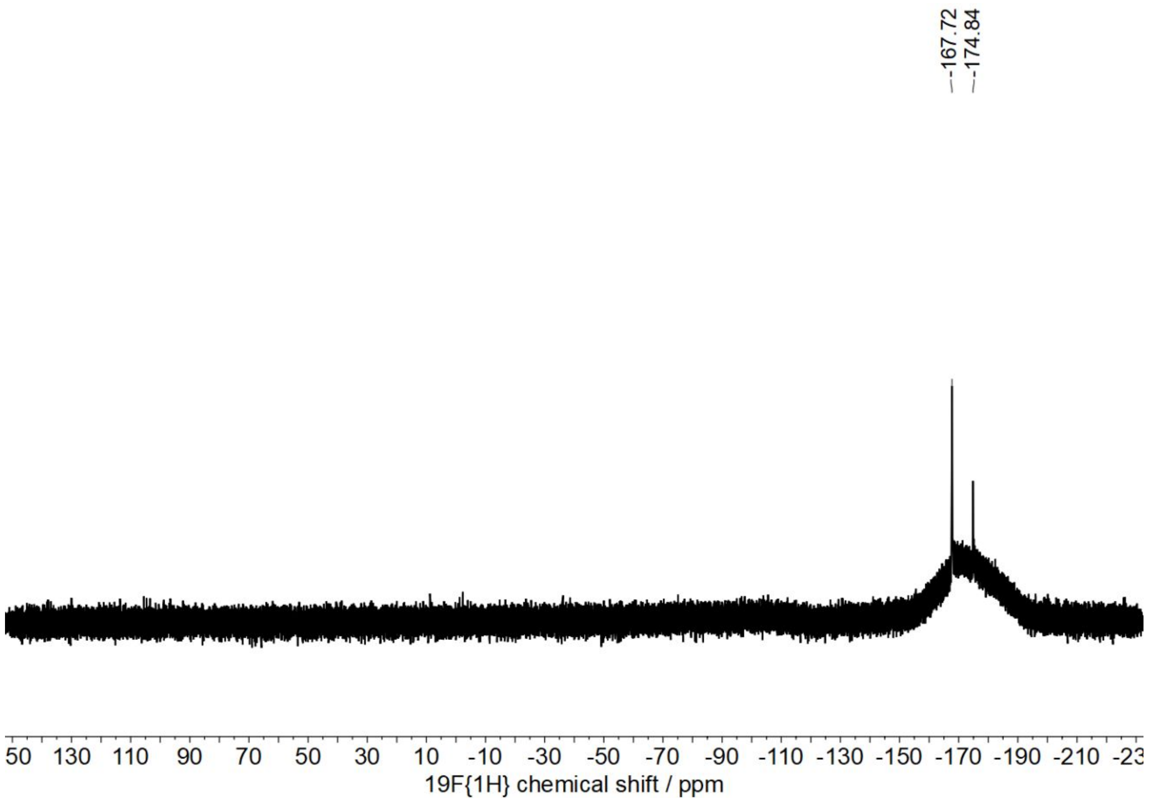

Figure S3. $^{19}F\{^{1}H\}$ NMR spectrum (565 MHz, C_6D_6, 293 K) of **2**.

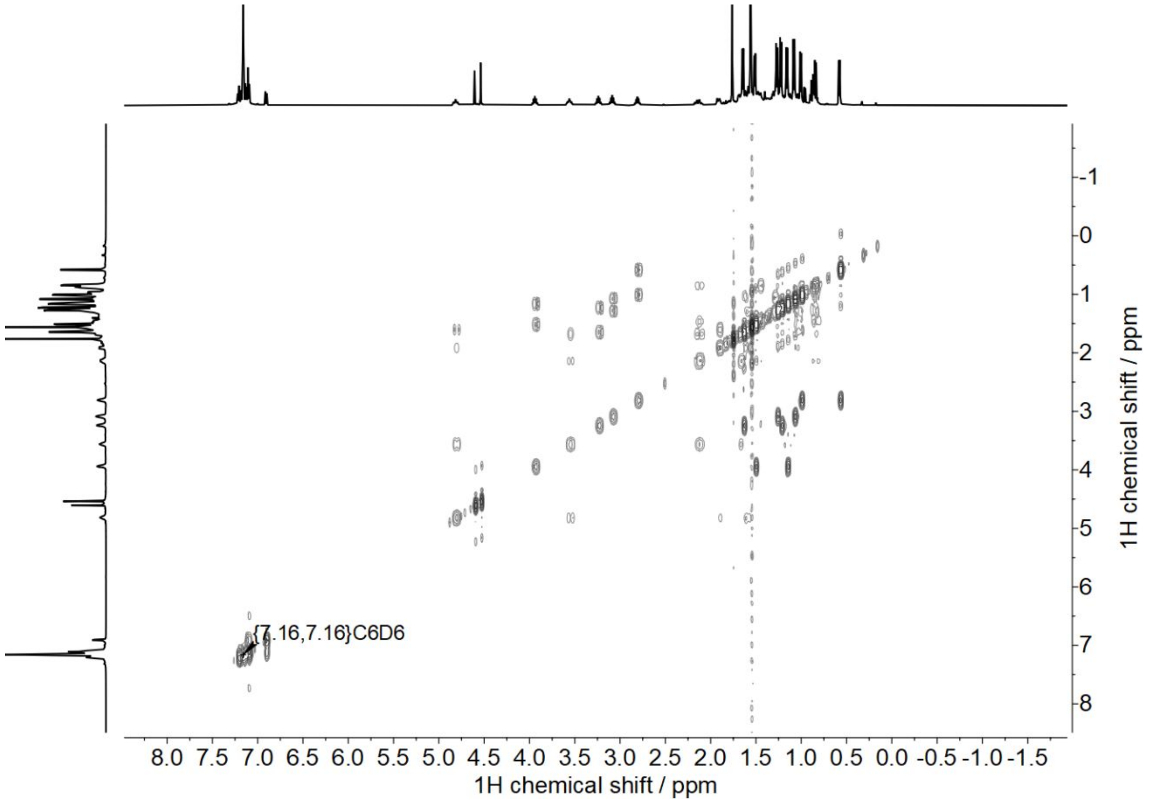

Figure S4. COSY NMR spectrum (500 MHz, C_6D_6, 293 K) of **2**.

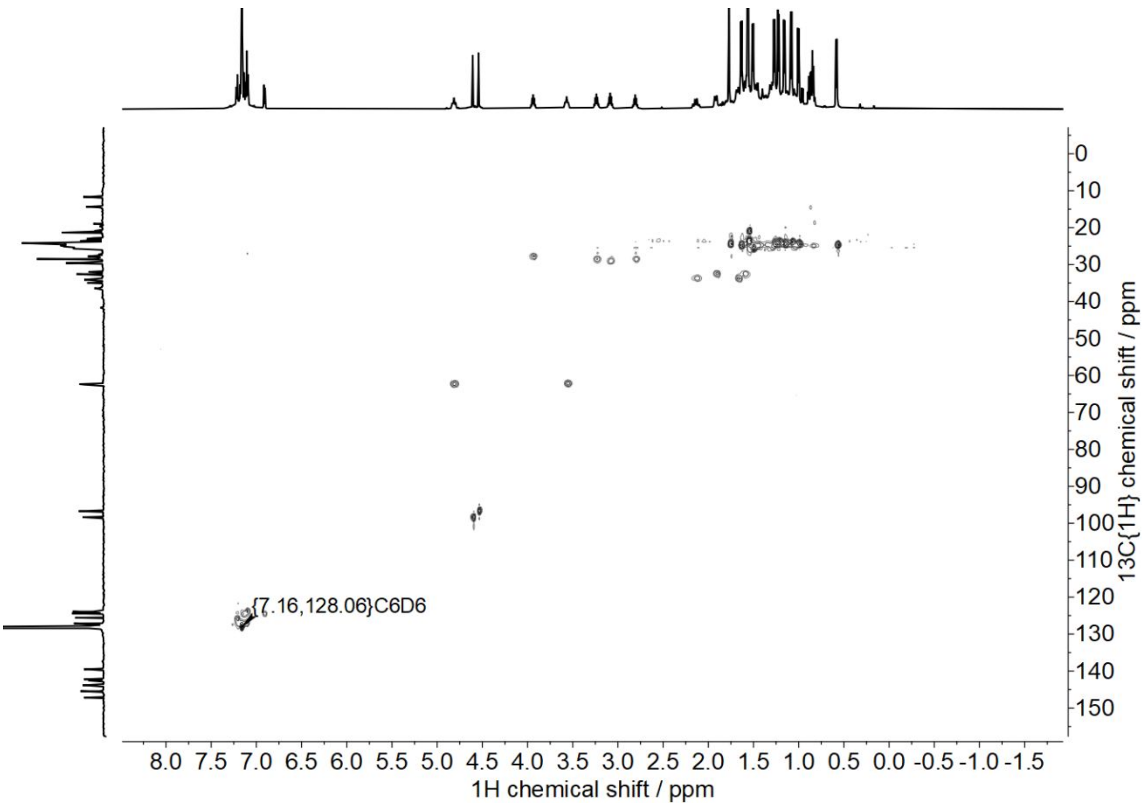

Figure S5. ^{1}H-$^{13}C\{^{1}H\}$ NMR spectrum (500 MHz, C_6D_6, 293 K) of **2**.

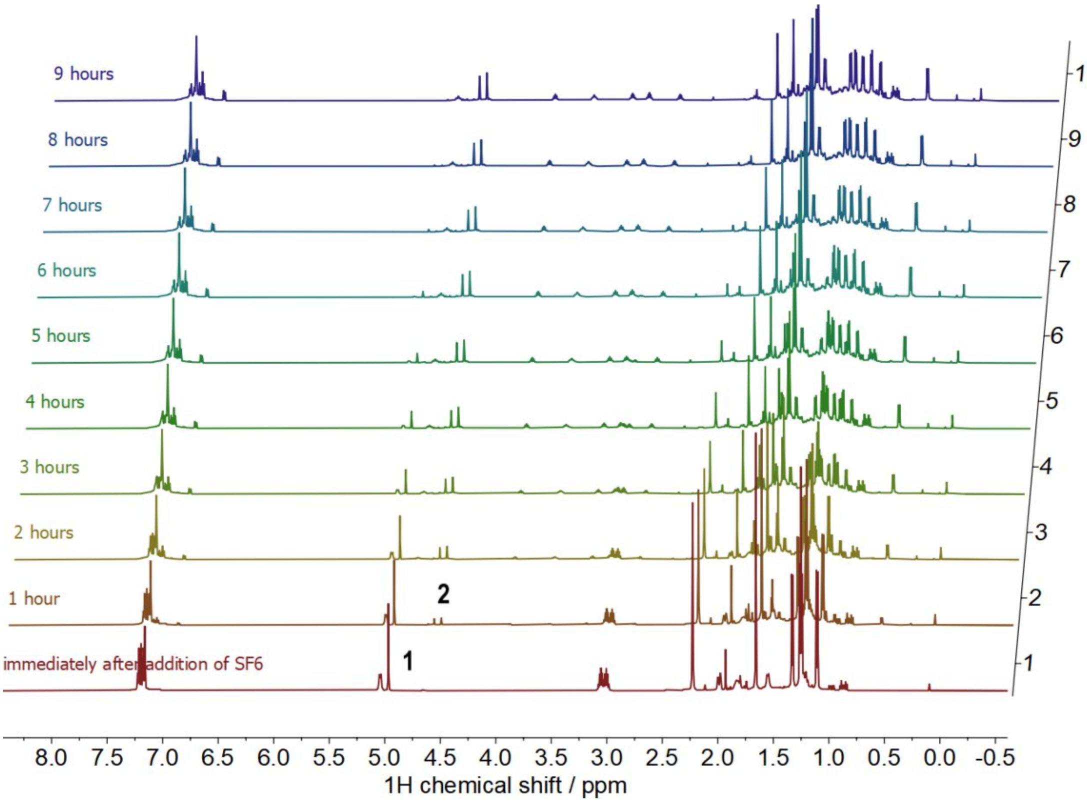

Figure S6. Series of ^{1}H NMR spectra taken at different time from the reaction mixture of **1** and SF_6; 20 mg (0.027 mmol) of **1** was dissolved in 0.4 mL of C_6D_6, degassed once via freeze-pump-thaw and SF_6 was added and heated to 80 °C.

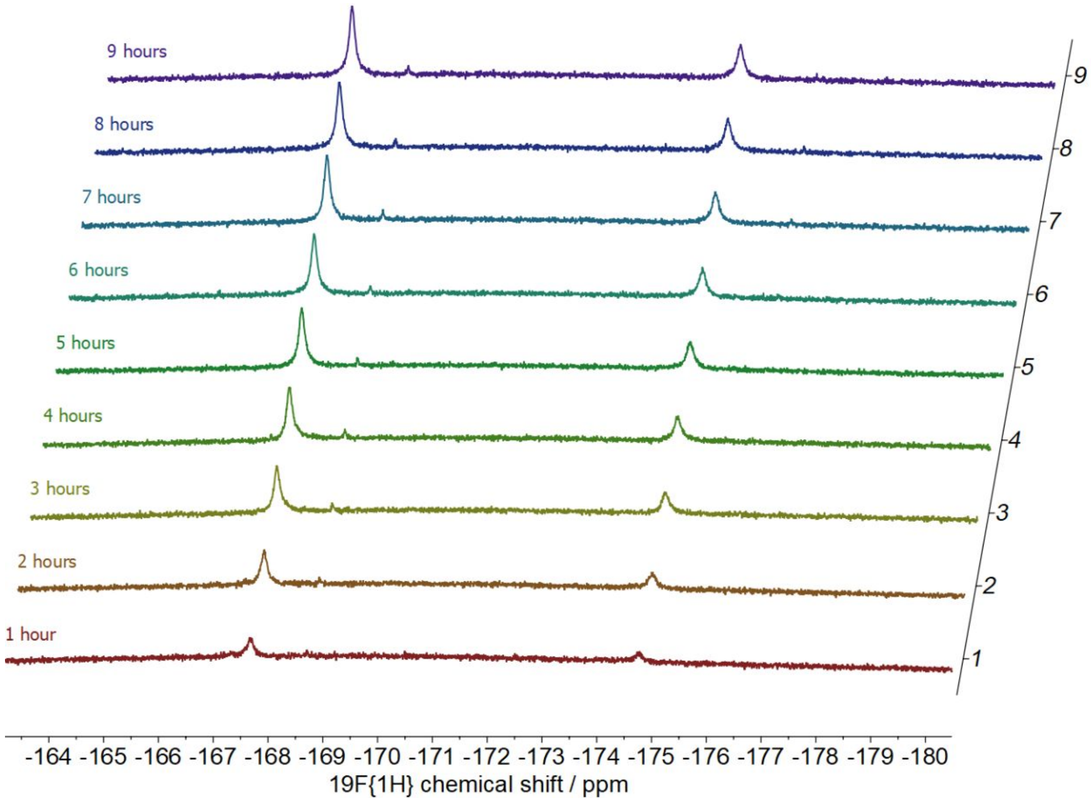

Figure S7. Series of $^{19}F\{^1H\}$ NMR spectra taken at different time from the reaction mixture of **1** and SF_6; 20 mg (0.027 mmol) of **1** was dissolved in 0.4 mL of C_6D_6, degassed once via freeze-pump-thaw and SF_6 was added and heated to 80 °C.

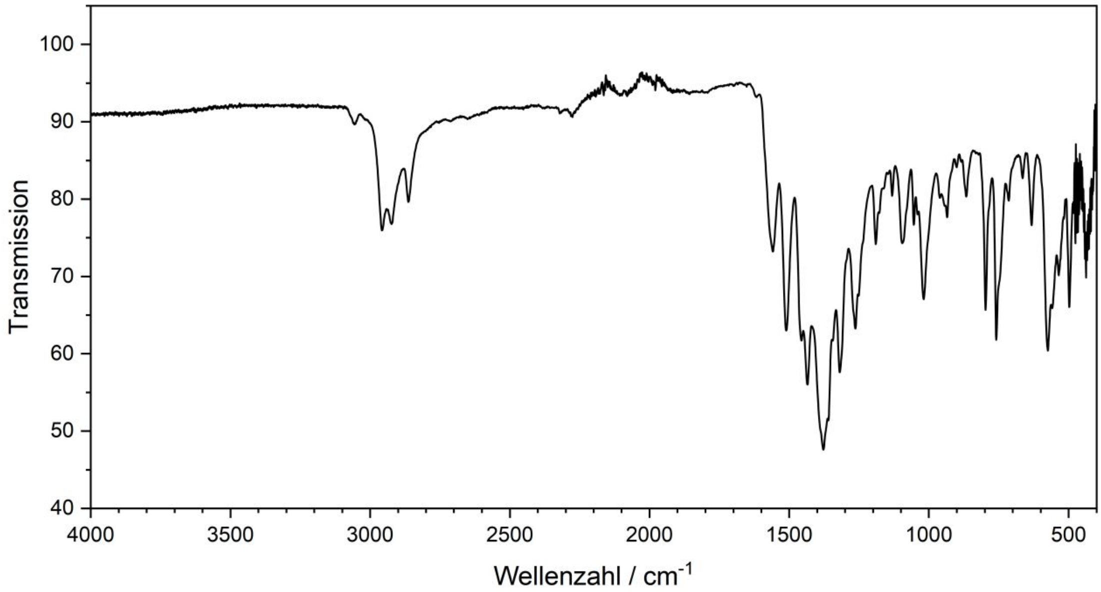

Figure S8. ATR-IR (diamond) spectrum of **2** indicating absence of characteristic bands of GaOH (around 3700 cm^{-1}) and GaH (around 1900 cm^{-1})[S5].

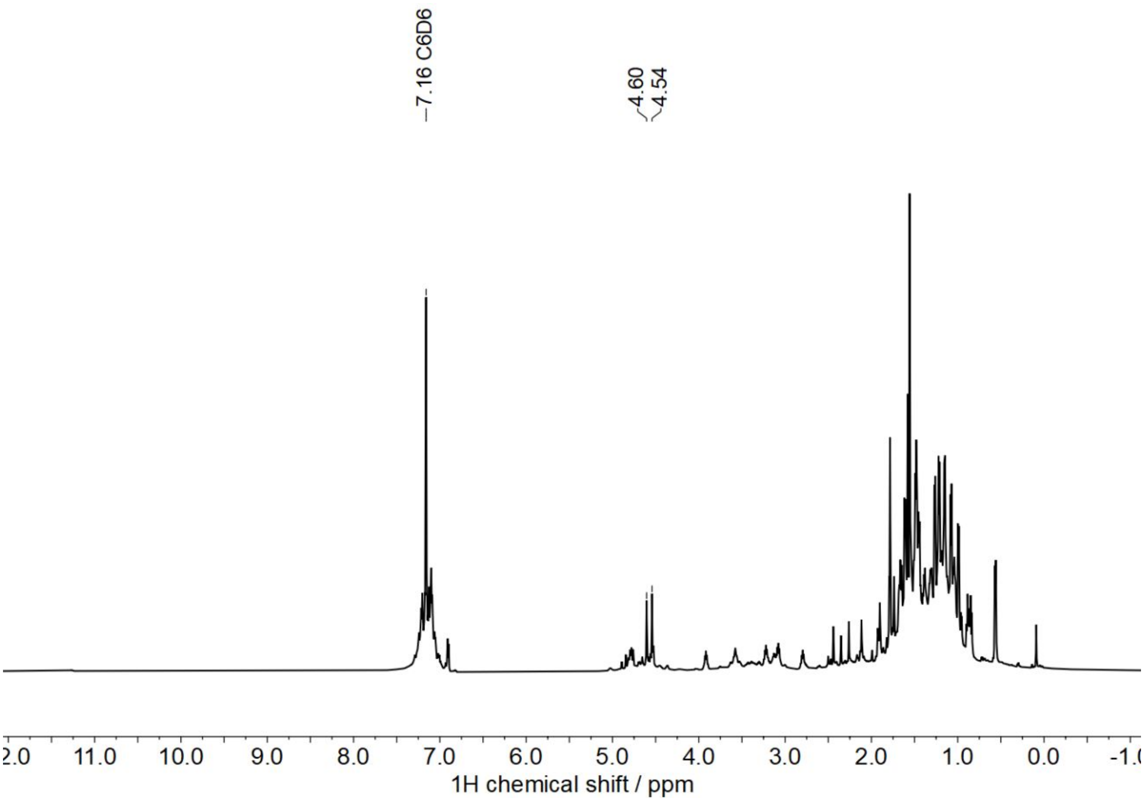

Figure S9. Crude ^{1}H NMR spectrum (500 MHz, C_6D_6, 293 K) of the reaction of **1** with SF_6 using a mercury arc lamp after six hours.

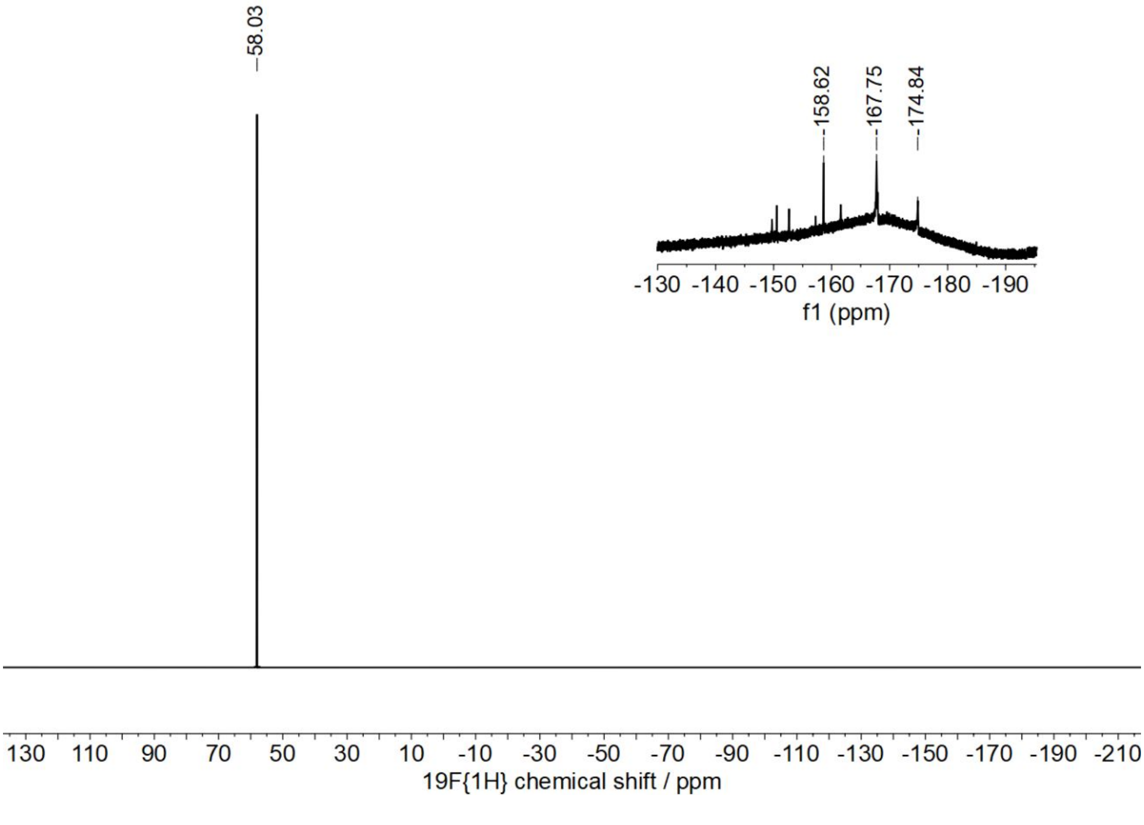

Figure S10. Crude $^{19}F\{^1H\}$ NMR spectrum (565 MHz, C_6D_6, 293 K) of the reaction of **1** with SF_6 using a mercury arc lamp after six hours.

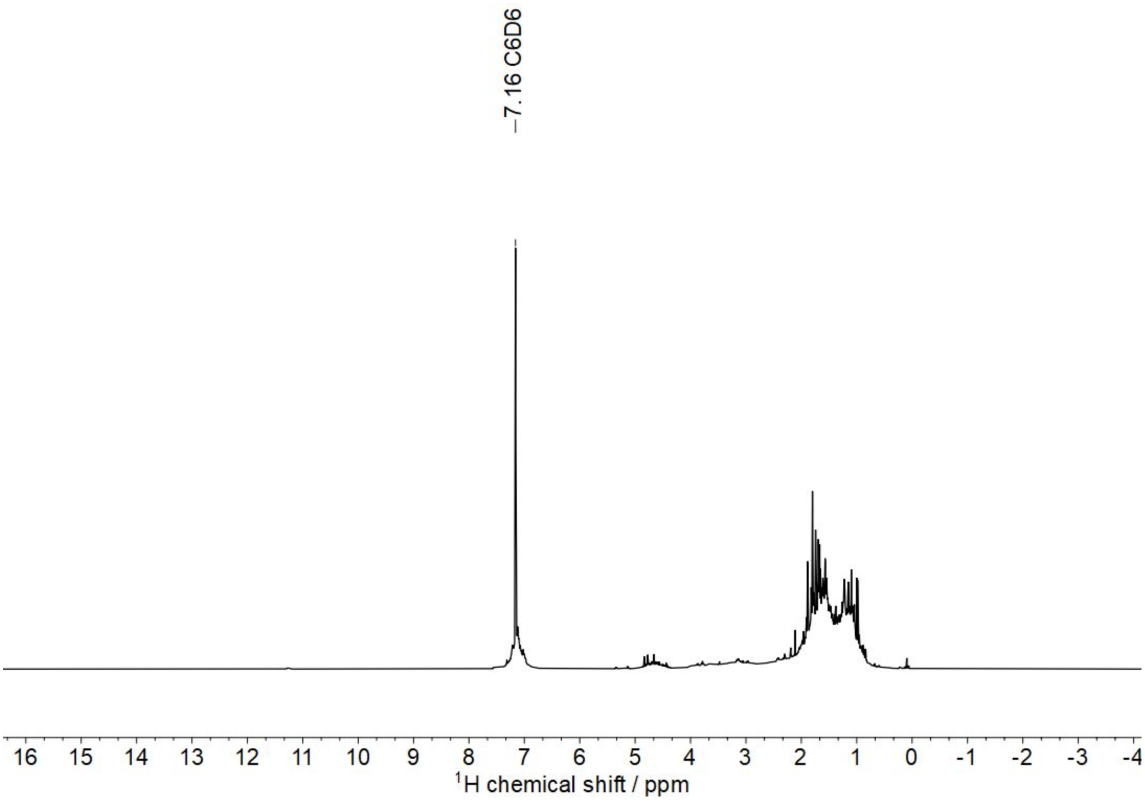

Figure S11. Crude ^{1}H NMR spectrum (500 MHz, C_6D_6, 293 K) of the reaction of **1** with S_8 after 16 hours.

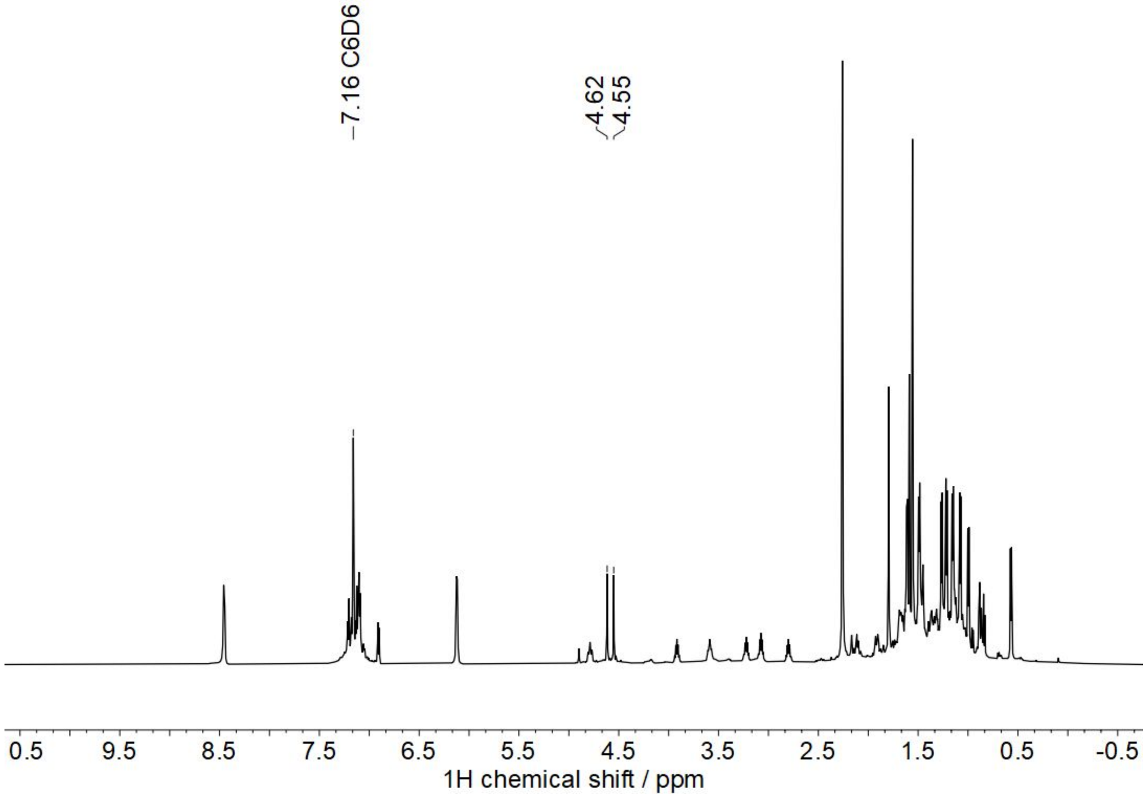

Figure S12. Crude ^{1}H NMR spectrum (500 MHz, C_6D_6, 293 K) of the reaction of **1** with SF_6 in the presence of 1.0 eq. 4-DMAP after 16 hours.

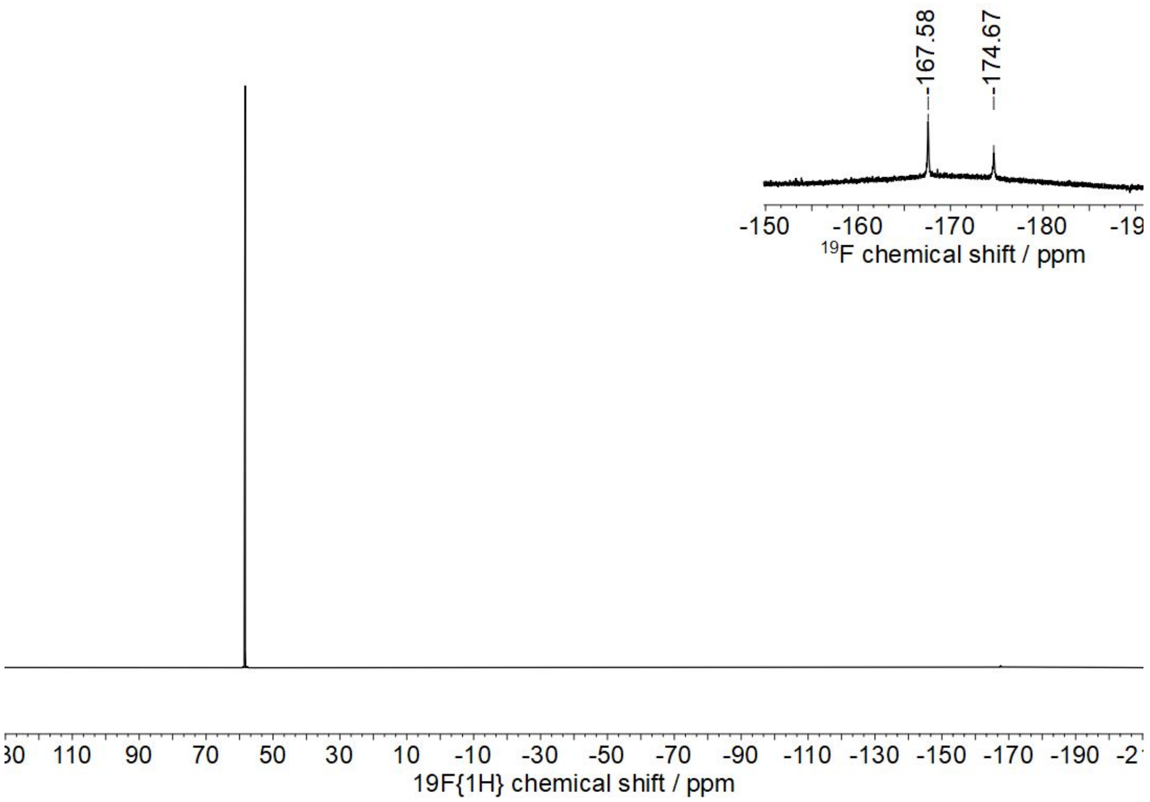

Figure S13. Crude $^{19}F\{^{1}H\}$ NMR spectrum (565 MHz, C_6D_6, 293 K) of the reaction of **1** with SF_6 in the presence of 1.0 eq. 4-DMAP after 16 hours.

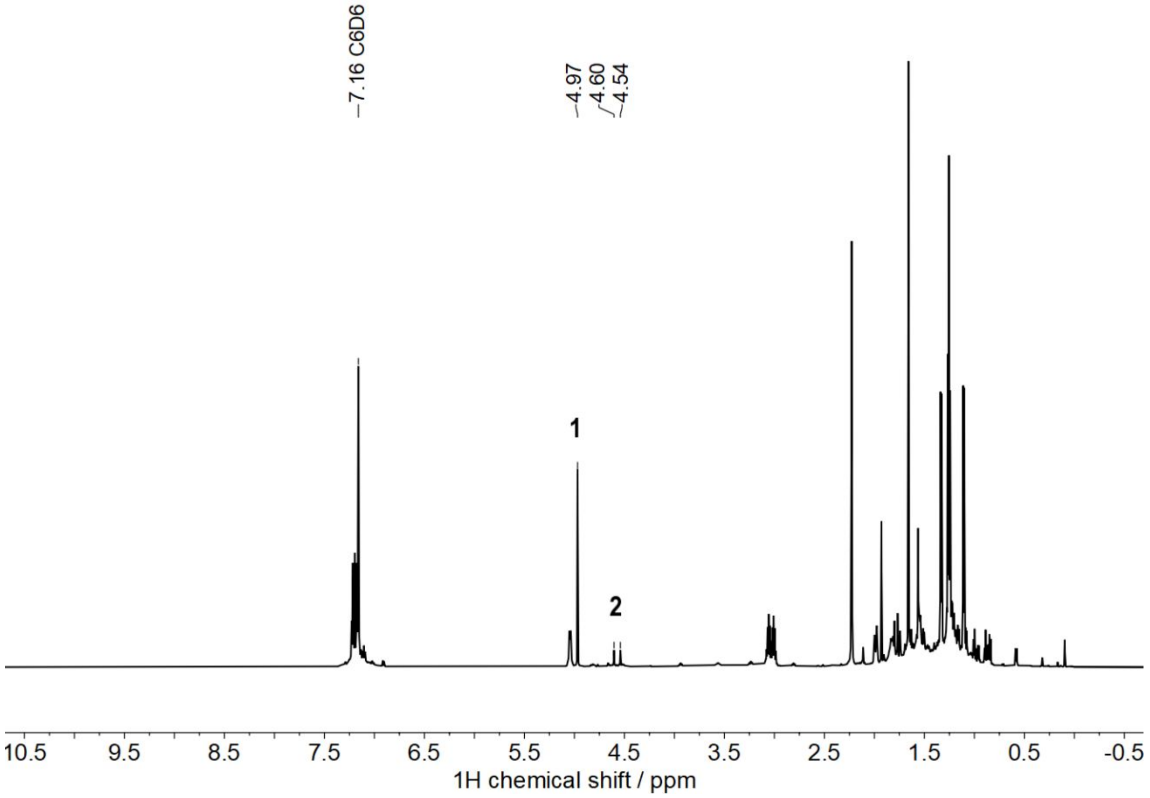

Figure S14. Crude ^{1}H NMR spectrum (500 MHz, C_6D_6, 293 K) of the reaction of **1** with 1.0 eq. SF_6 after 25.5 hours.

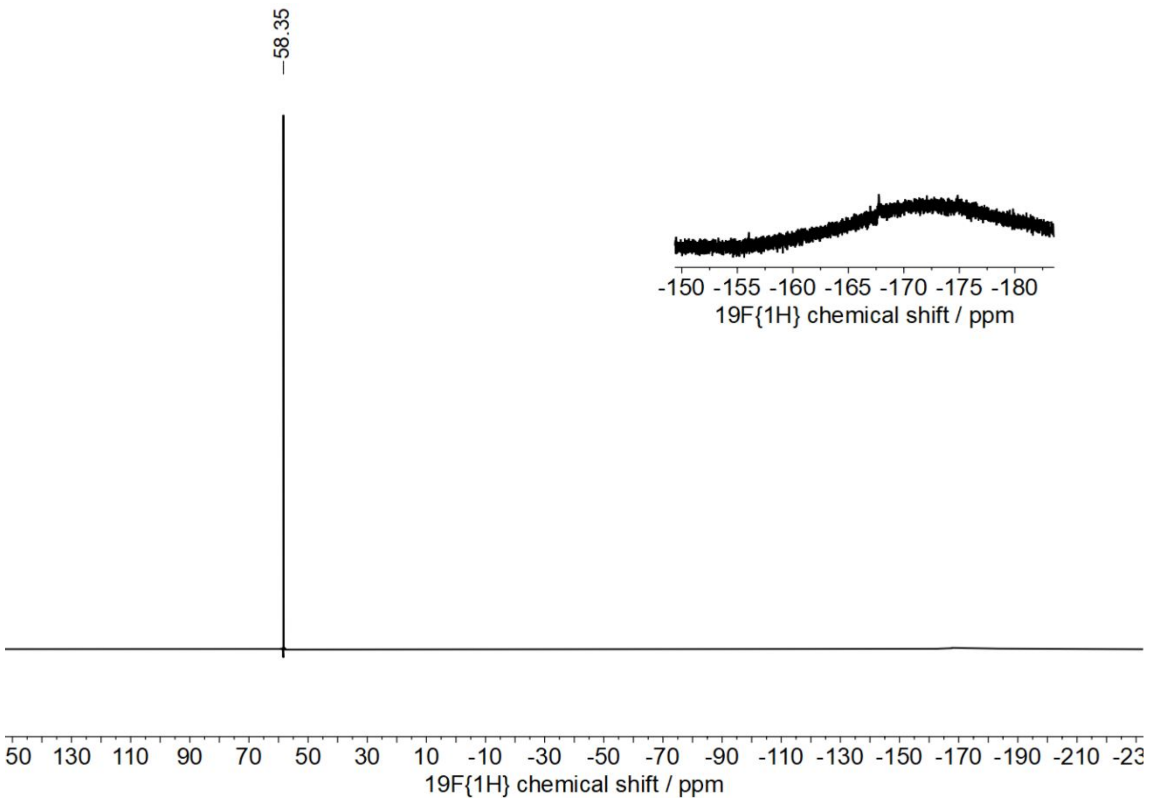

Figure S15. Crude $^{19}F\{^{1}H\}$ NMR spectrum (565 MHz, C_6D_6, 293 K) of the reaction of **1** with 1.0 eq. SF_6 after 25.5 hours.

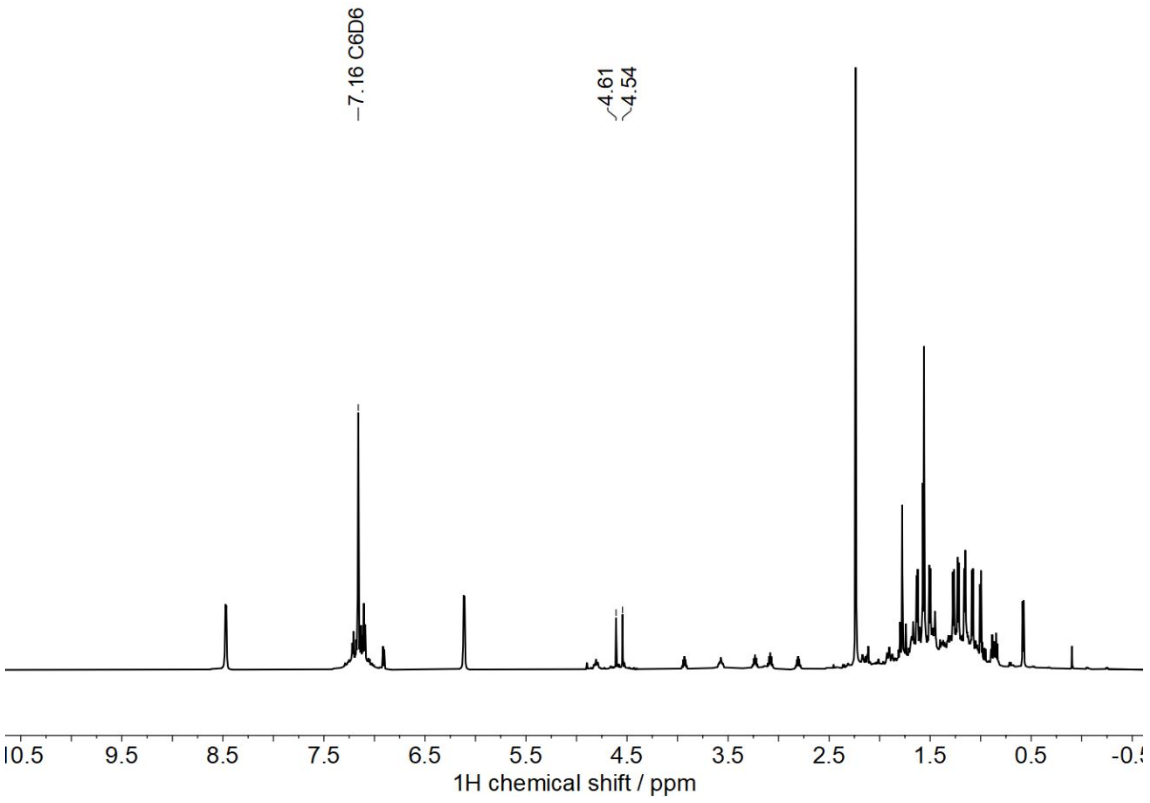

Figure S16. Crude ^{1}H NMR spectrum (500 MHz, C_6D_6, 293 K) of the reaction of **1** with 1.0 eq. SF_6 and in the presence of 1.0 eq. 4-DMAP after 24 hours.

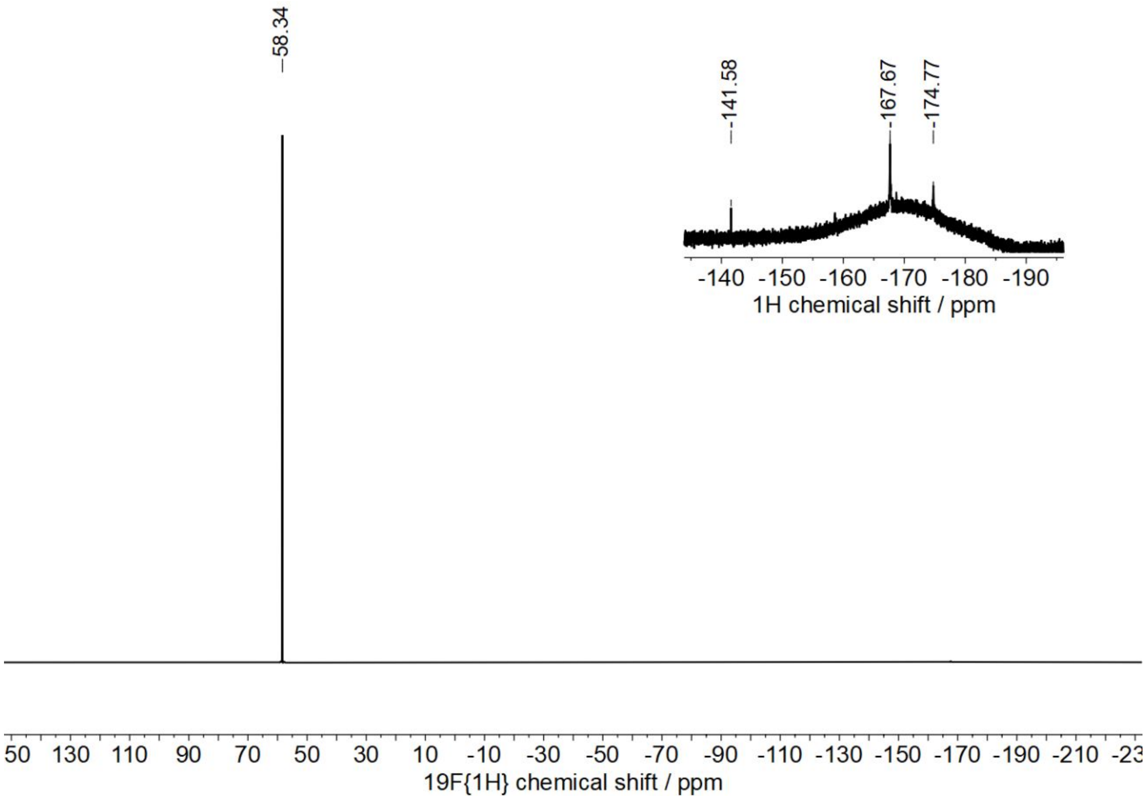

Figure S17. Crude $^{19}F\{^{1}H\}$ NMR spectrum (565 MHz, C_6D_6, 293 K) of the reaction of **1** with 1.0 eq. SF_6 and in the presence of 1.0 eq. 4-DMAP after 24 hours; a new signal at –141.6 ppm could observed while the reaction solution was red, in the yellow solution after the reaction was finished the signal couldn't be observed anymore.

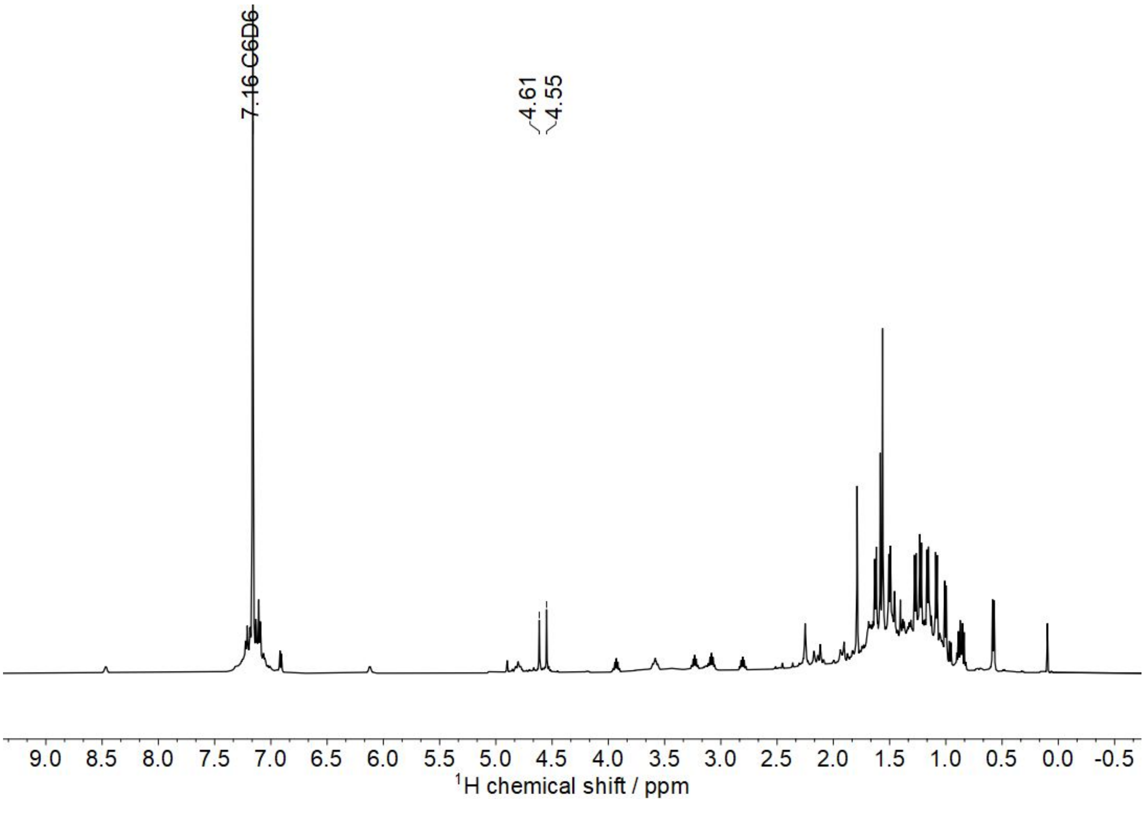

Figure S18. Crude ^{1}H NMR spectrum (500 MHz, C_6D_6, 293 K) of the reaction of **1** with SF_6 in the presence of 0.1 eq. 4-DMAP after 48 hours.

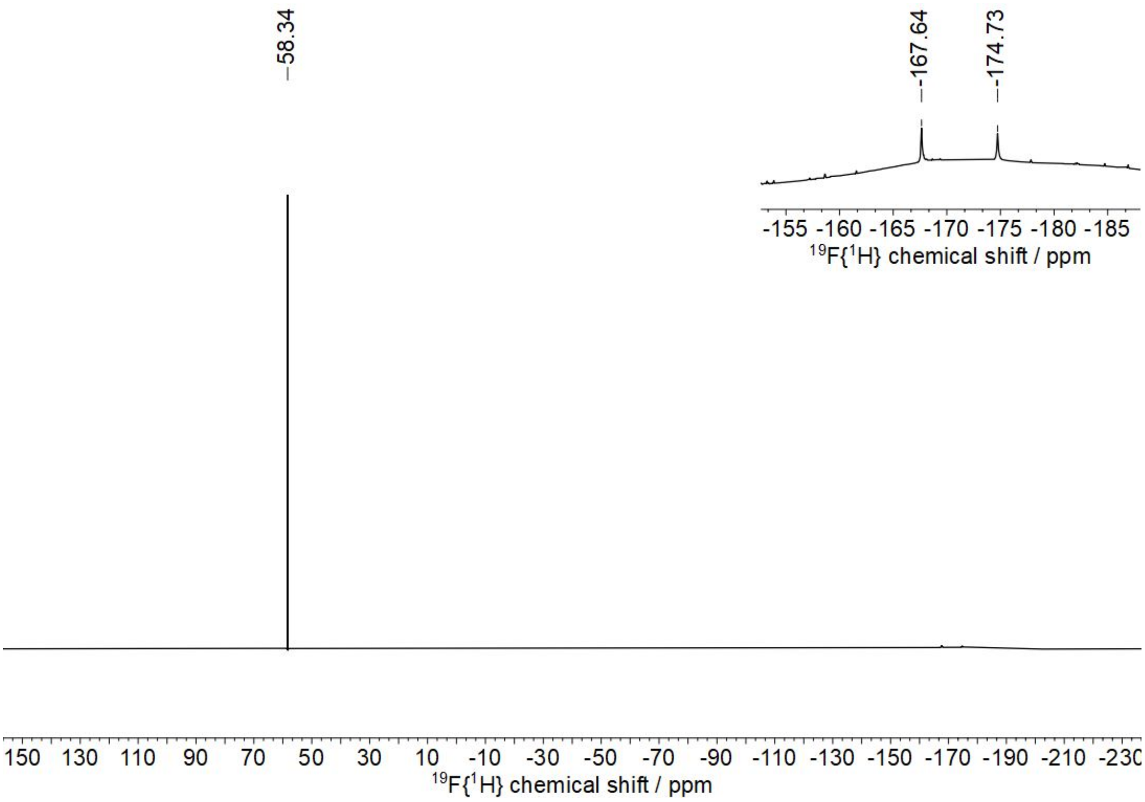

Figure S19. Crude $^{19}F\{^{1}H\}$ NMR spectrum (565 MHz, C_6D_6, 293 K) of the reaction of **1** with SF_6 in the presence of 0.1 eq. 4-DMAP after 48 hours.

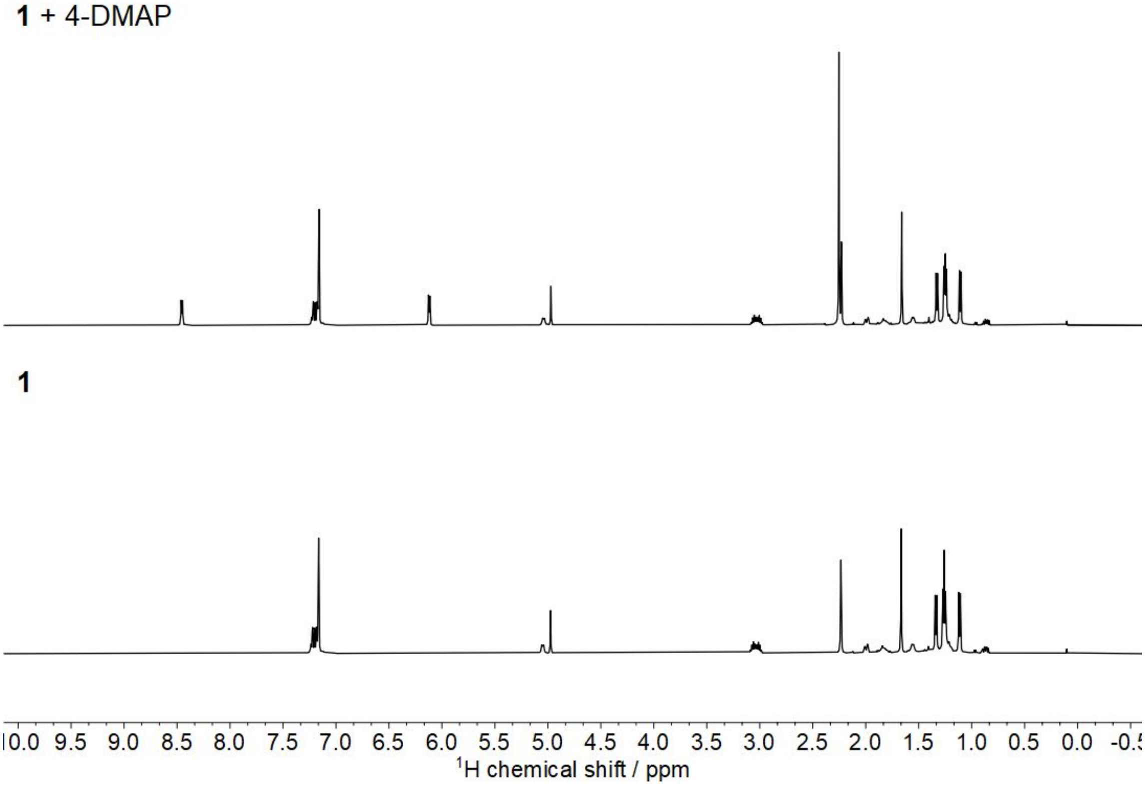

Figure S20. ^{1}H NMR spectra (500 MHz, C_6D_6, 293 K) of **1** and of the reaction of **1** with 1.0 eq. 4-DMAP.

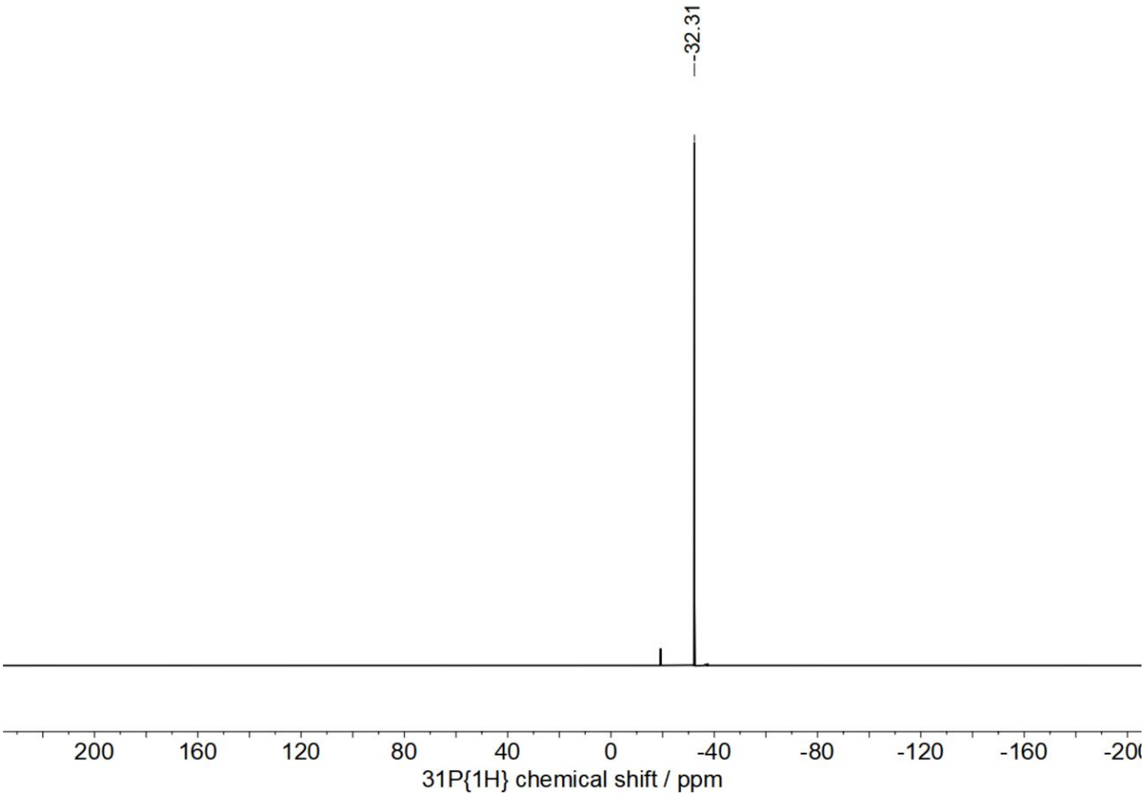

Figure S21. $^{31}P\{^{1}H\}$ NMR spectrum (203 MHz, C_6D_6, 293 K) after addition of tri-*n*-butylphosphine to the reaction solution after the reaction was finished.

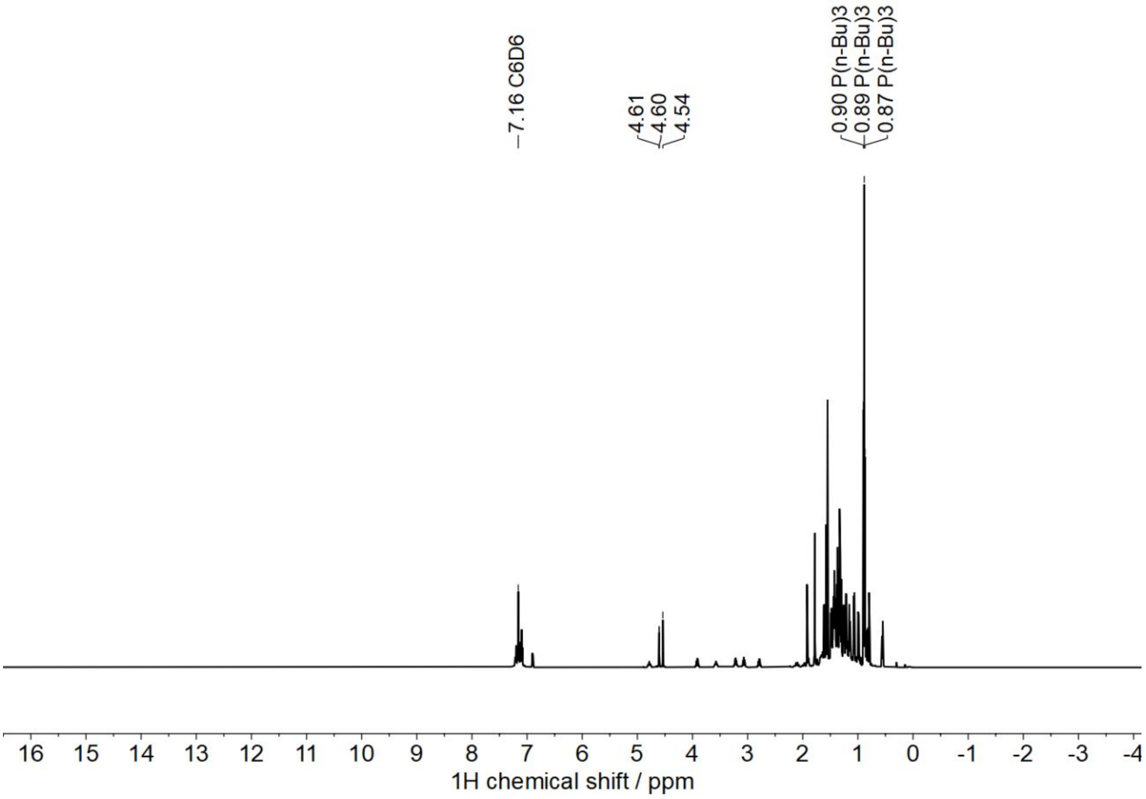

Figure S22. ^{1}H NMR spectrum (500 MHz, C_6D_6, 293 K) of the reaction of **1** with SF_6 in the presence of tri-*n*-butylphosphine after 17 hours at 80 °C.

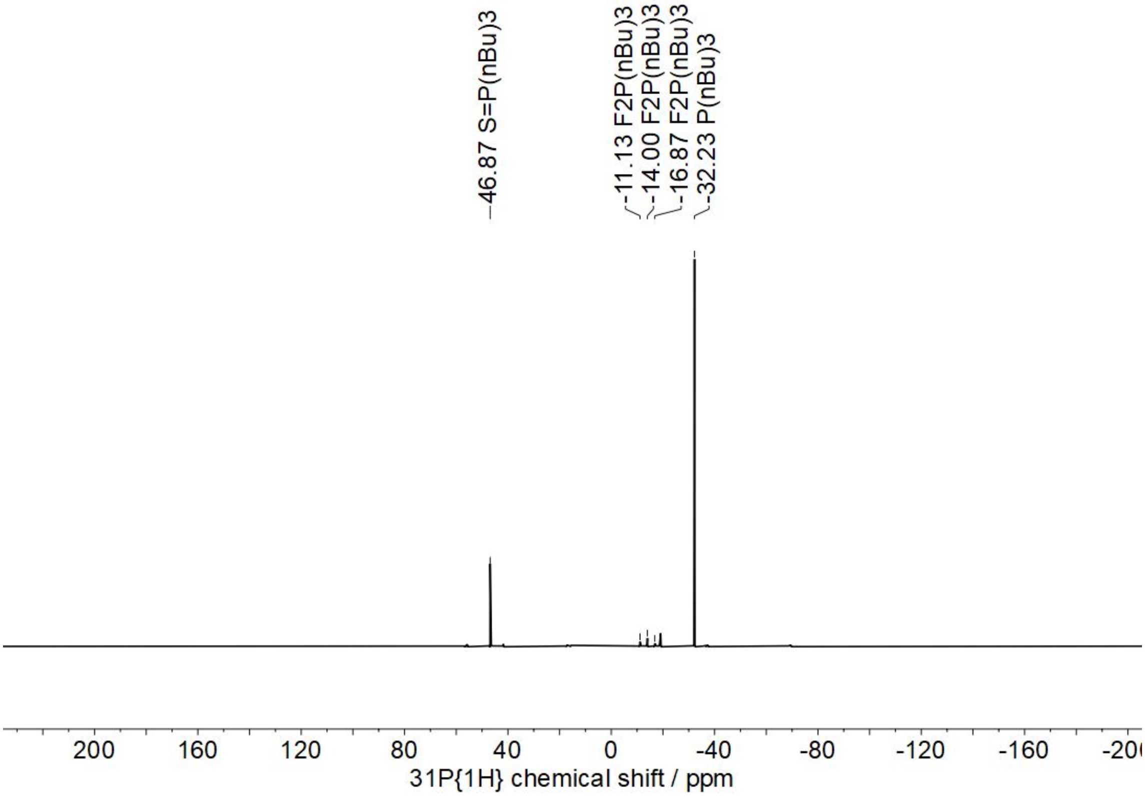

Figure S23. $^{31}P\{^{1}H\}$ NMR spectrum (203 MHz, C_6D_6, 293 K) of the reaction of **1** with SF_6 in the presence of tri-*n*-butylphosphine after 17 hours at 80 °C.

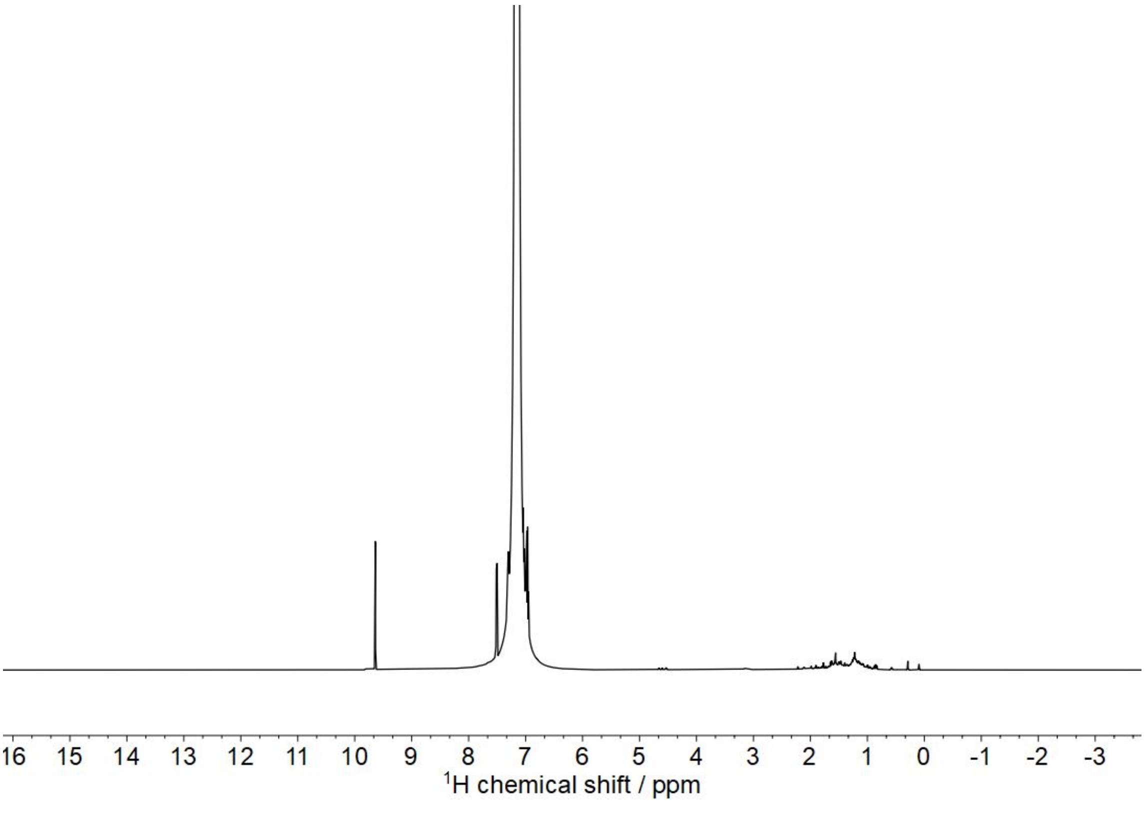

Figure S24. ^{1}H NMR spectrum (500 MHz, C_6D_6, 293 K) after addition of benzaldehyde to the reaction solution after the reaction was finished. The compound **2** was completely decomposed.

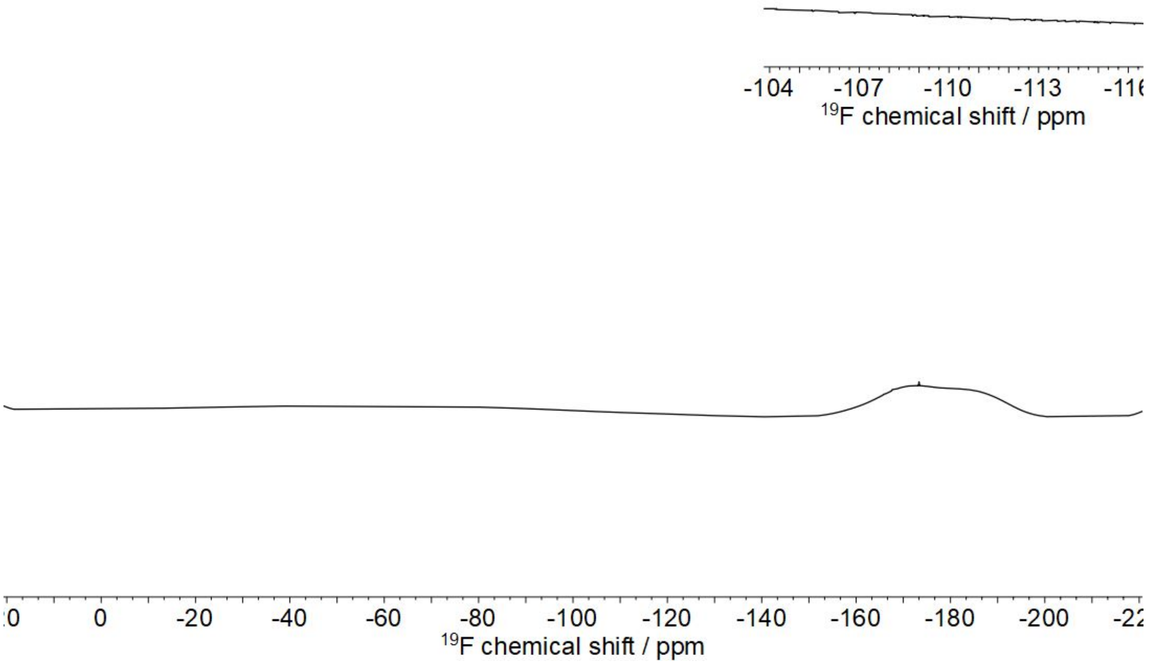

Figure S25. ^{19}F NMR spectrum (471 MHz, C_6D_6, 293 K) after addition of benzaldehyde to the reaction solution after the reaction was finished. No signal for (difluoromethyl)benzene at –110.7 ppm[S6] could be observed as expected for the reaction of possibly formed SF_4 with benzaldehyde.[S7]

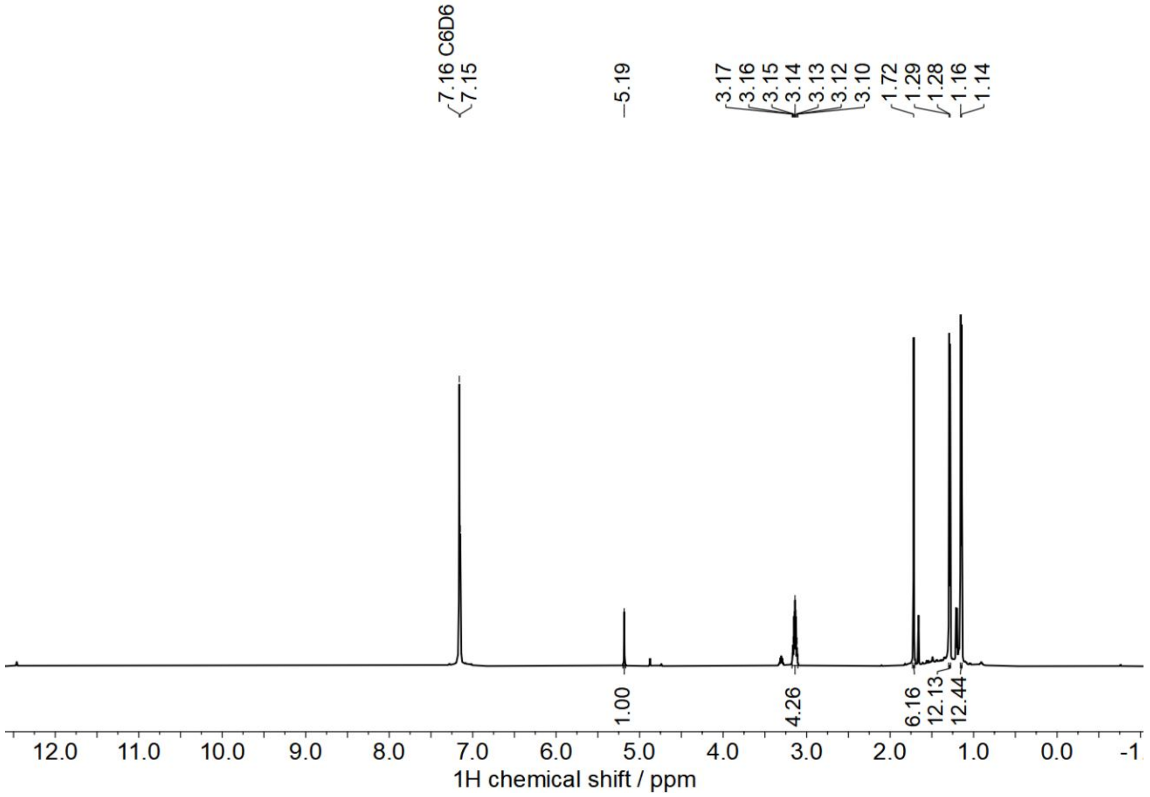

Figure S26. ^1H NMR spectrum (500 MHz, C_6D_6, 293 K) of the reaction of **3** with SF_6 at 80°C; only the resonances for **3** were observed, according to the literature[S8].

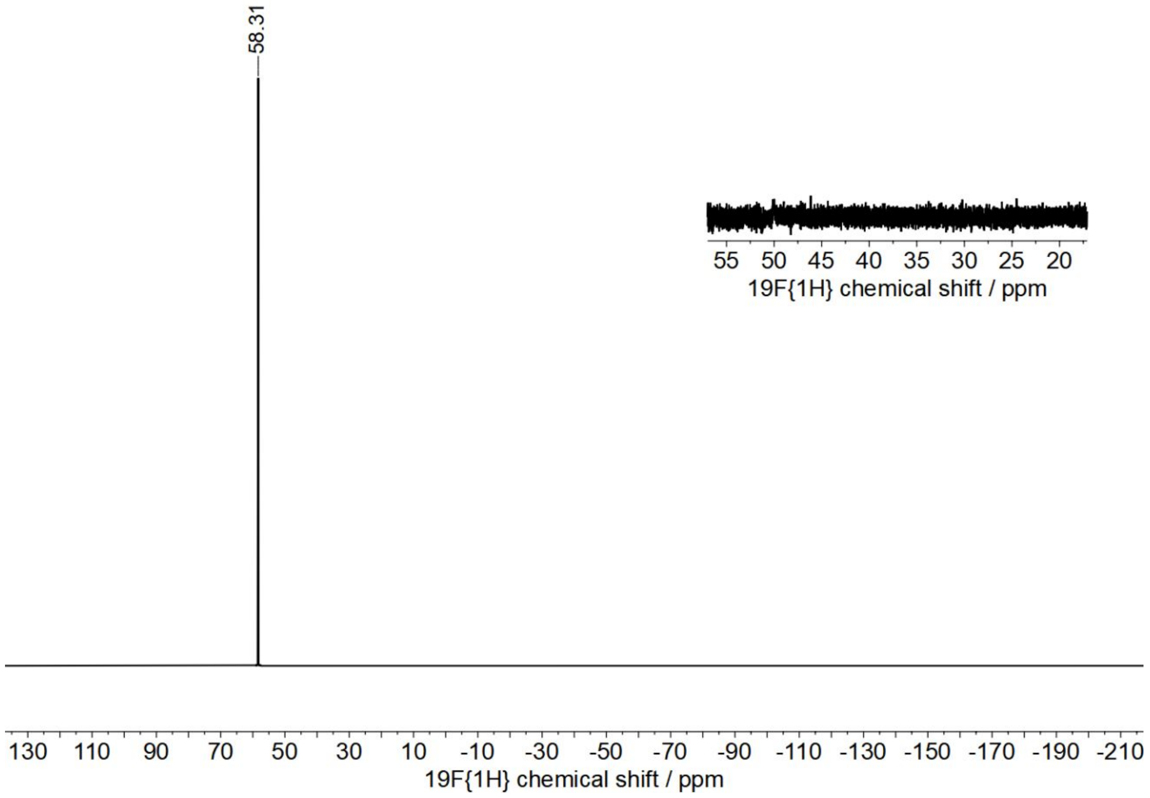

Figure S27. $^{19}F\{^1H\}$ NMR spectrum (565 MHz, C_6D_6, 293 K) of the reaction of **3** with SF_6 at 80°C; only the resonance for SF_6 were observed and no resonance for **4** at 38.34 ppm[S9].

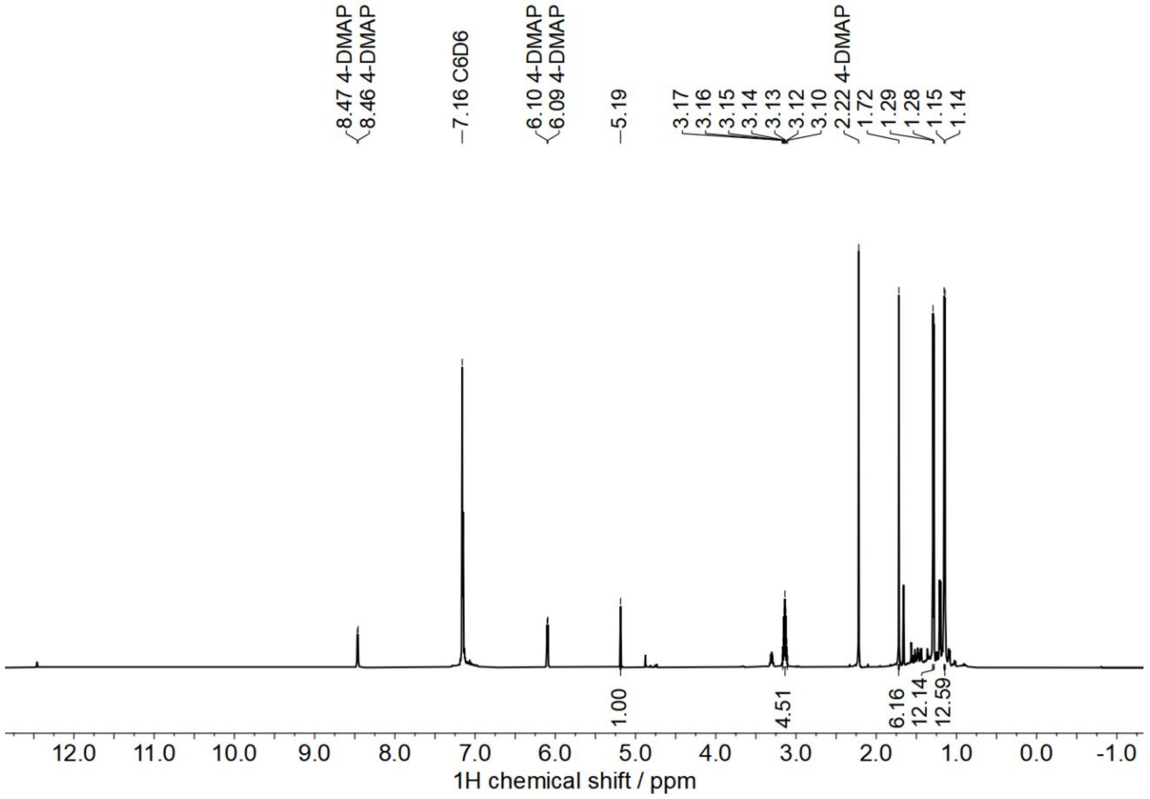

Figure S28. ^1H NMR spectrum (500 MHz, C_6D_6, 293 K) of the reaction of **3** with SF_6 and in the presence of 4-DMAP after 16 hours at 80°C; only the resonances for **3** were observed, according to the literature[S8].

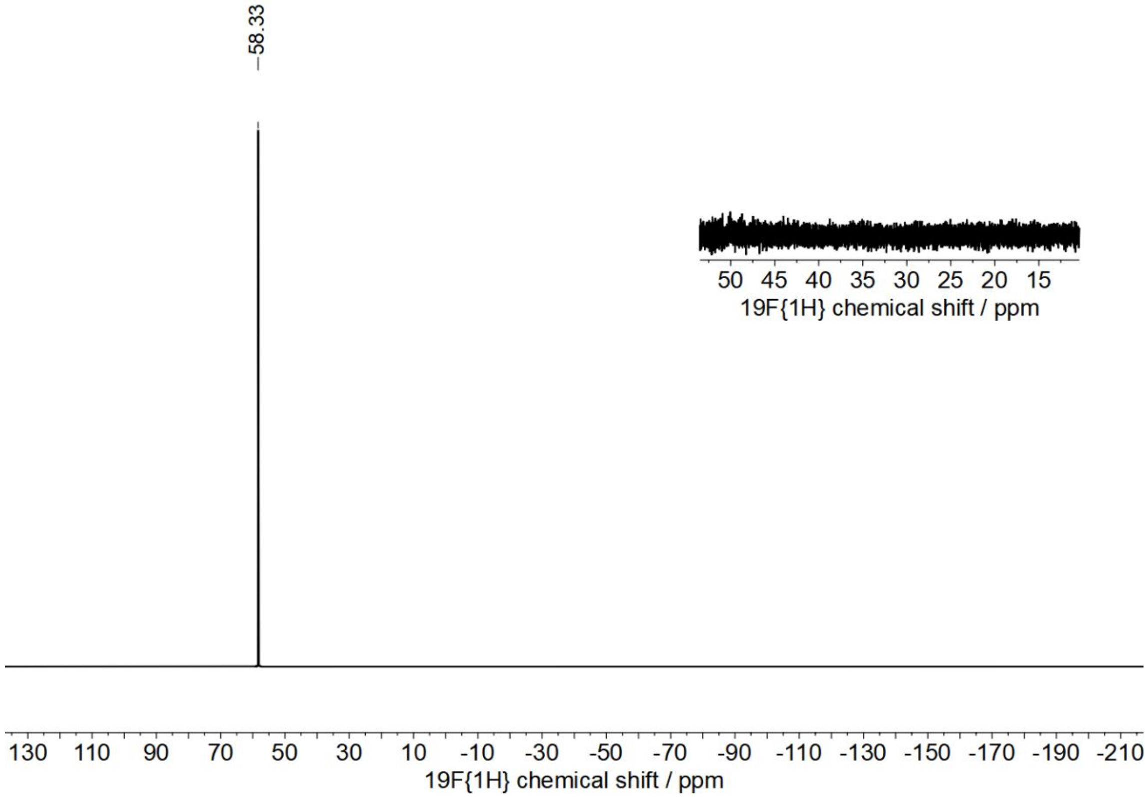

Figure S29. $^{19}F\{^{1}H\}$ NMR spectrum (565 MHz, C_6D_6, 293 K) of the reaction of **3** with SF_6 and in the presence of 4-DMAP after 16 hours at 80°C; only the resonance for SF_6 were observed and no resonance for **4** at 38.34 ppm[S9].

3. Computational Details

3.1. Bonding Parameters Comparison to XRD Data

Table S3. Important bonding parameters of XRD structure **2** with the computationally obtained structure. All bond distances are in Angstroms (Å) and degrees (°) for bond angles. For the M06-2X computationally obtained structure, all distances and angles were obtained through the Avogadro 2 (version 1.101.0) graphical user interface.[S10]

Bond distance and angles	XRD	M06-2X
Ga1–Ga2	2.4115(6)	2.494
Ga1–F1	1.8204(18)	1.817
Ga1–N1	1.988(2)	2.021
Ga1–N2	1.939(2)	1.970
Ga2–F2	1.8054(17)	1.807
Ga2–N3	1.988(2)	1.997
Ga2–N4	1.941(2)	1.957
N1–Ga1–N2	97.34(9)	96.379
N3–Ga2–N4	96.18(9)	93.998

3.2. Energy Decomposition Analysis Data

All EDA-NOCV computations were computed at the M06-2X/TZP[S11,S12] level of theory using the AMS 2024.102 software.[S13] Relaxed fragments were obtained via geometry optimization in AMS at the same level of theory, while frozen fragments were taken from prior optimizations. See the computations section of the manuscript for further details.

Table S4. EDA-NOCV results at the M06-2X/TZP level of theory. All energies are given in kJ·mol^{-1}.

ΔE_{int}	-29.0
ΔE_{Pauli}	134.8
ΔE_{elstat}	-114.2 (70%)[a]
ΔE_{orb}	-49.6 (30%)[a]
ΔE_1	-15.3 (31%)[b]
ΔE_{rest}	-34.3 (69%)[b]
ΔE_{prep}	17.9
E_{bond}	-11.1

[a] The percentage represents the contribution towards the total attractive interaction (i.e., $\Delta E_{elstat} + \Delta E_{orb}$).
[b] The energetic and percent contribution of the NOCV deformation densities towards the total ΔE_{orb}.

The EDA scheme used in this works follows that of Morokuma,[S14] as well as Ziegler and Rauk.[S15,S16] This scheme separates the bonding energy into two components: preparation energy (ΔE_{prep}) and the instantaneous interaction energy (ΔE_{int}).

$$E_{\text{bond}} = \Delta E_{\text{int}} + \Delta E_{\text{prep}}$$

The interaction energy is computed as the sum of the Pauli repulsion energy (ΔE_{Pauli}), quassiclassical electrostatic interaction energy (ΔE_{elstat}), and orbital mixing and relaxation energy (ΔE_{orb}) terms.

$$\Delta E_{\text{int}} = \Delta E_{\text{Pauli}} + \Delta E_{\text{elstat}} + \Delta E_{\text{orb}}$$

The preparation energy is the energy required to deform the isolated fragments A and B from their optimized geometries and ground-state electronic configurations to those adopted in the final complex AB.

$$\Delta E_{\text{prep}} = E_A^{\text{frozen}} - E_A^{\text{GS}} + E_B^{\text{frozen}} - E_B^{\text{GS}}$$

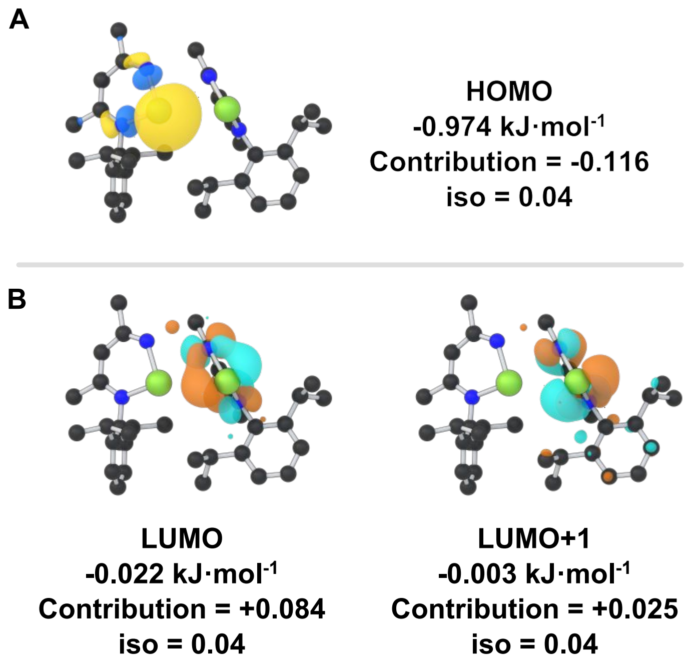

Figure S30. (**A**) SFO HOMO contribution to the Ga[1] fragment indicating electron density loss. (**B**) SFO LUMO and LUMO+1 contribution to the Ga[2] fragment indicating electron density gain. Only SFO contributions >0.01 were represented in the decomposition of $\Delta\rho_1$.

3.3. Nudged Elastic Band Minimum Energy Pathways

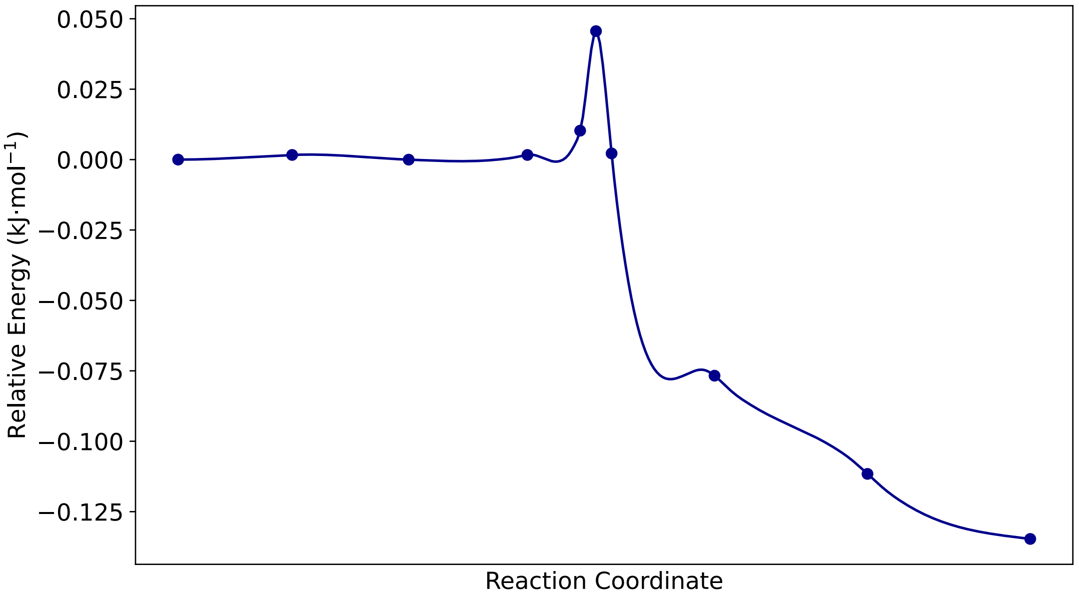

Figure S31. Final MEP of **1a** (left) towards **Int1** (right) using the nudged elastic band approach. The calculation was conducted at the M06-2X/def2-TZVP level of theory and all energies are provided in kJ·mol-1.

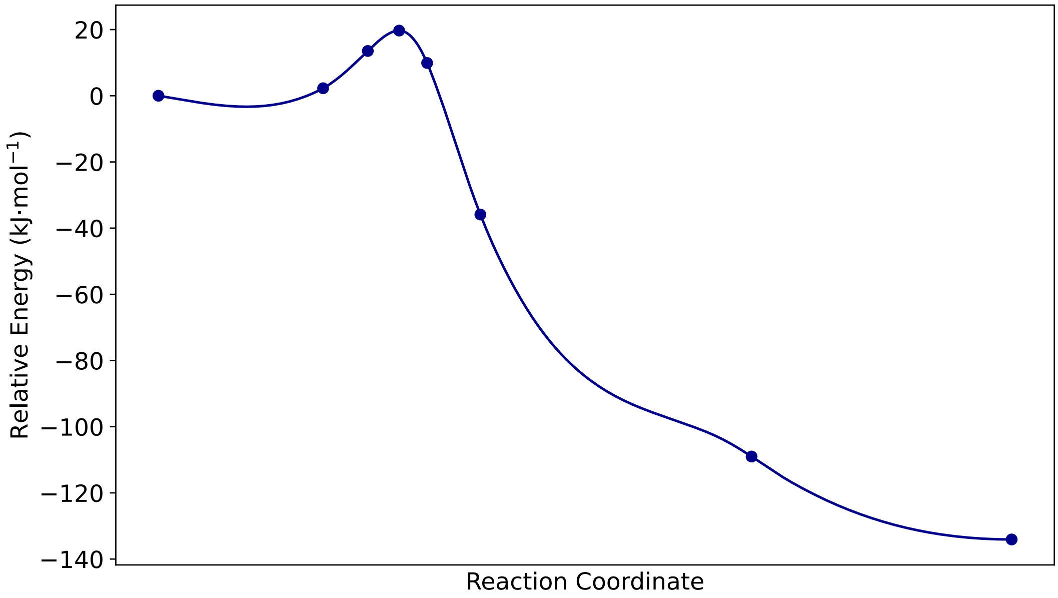

Figure S32. Final MEP of **Int1** (left) towards **2a** (right) using the nudged elastic band approach. The calculation was conducted at the M06-2X/def2-TZVP level of theory and all energies are provided in kJ·mol^{-1}.

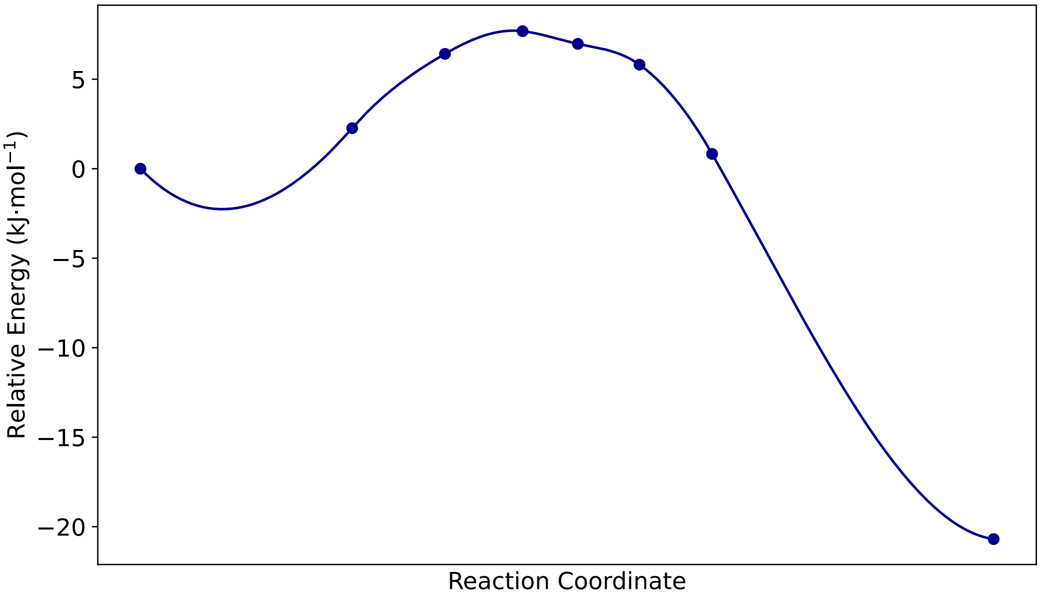

Figure S33. Final MEP of **1b** (left) towards **Int2** (right) using the nudged elastic band approach. The calculation was conducted at the M06-2X/def2-TZVP level of theory and all energies are provided in kJ·mol^{-1}.

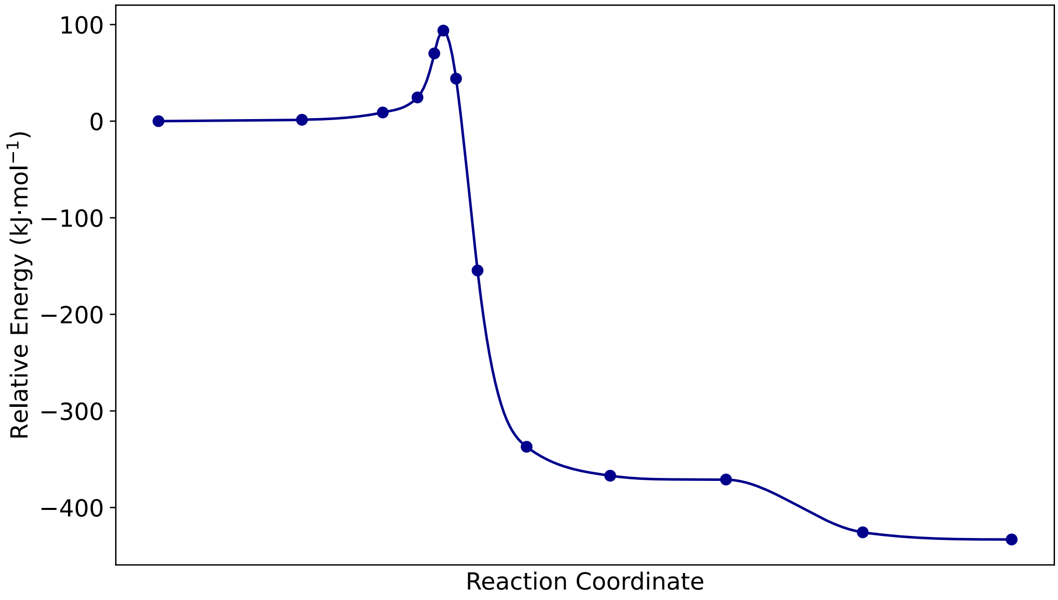

Figure S34. Final MEP of **Int2** (left) towards **Int3** (right) using the nudged elastic band approach. The calculation was conducted at the M06-2X/def2-TZVP level of theory and all energies are provided in kJ·mol^{-1}.

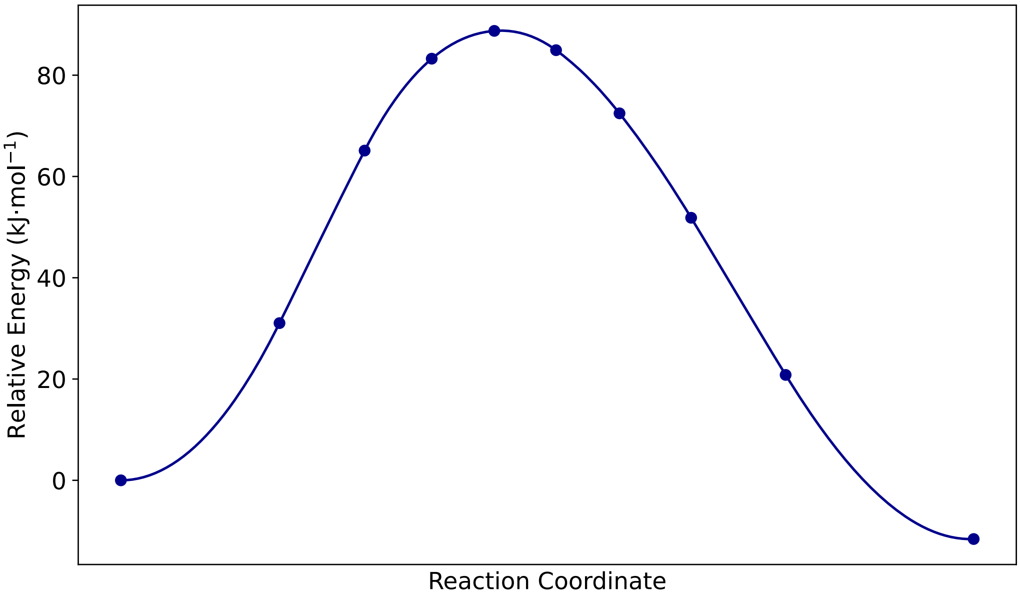

Figure S35. Final MEP of **Int3** (left) towards **2b** (right) using the nudged elastic band approach. The calculation was conducted at the M06-2X/def2-TZVP level of theory and all energies are provided in kJ·mol^{-1}.

3.4. Reductive Elimination Site Investigation

In order to assure accurate nudged elastic band MEPs, each of the reductive elimination fluorine site products (i.e., **2a**) were created using the Avogadro 2 (version 1.101.0) graphical user interface.[S10] Subsequently, the various **2a** variations were subjected to a geometry optimization using ORCA 6.0.0 at the M06-2X/def2-TZVP level of theory. The nudged elastic band approach was applied between **Int1** and the different **2a** variations to obtain the transition state structures of the various reductive elimination sites (**TS2a-d**). The activation energy barriers were then calculated using the DLPNO-CCSD(T)/def2-TZVPP level of theory while applying Gibbs free thermodynamic energy corrections from the previously mentioned DFT level at 298.15K.

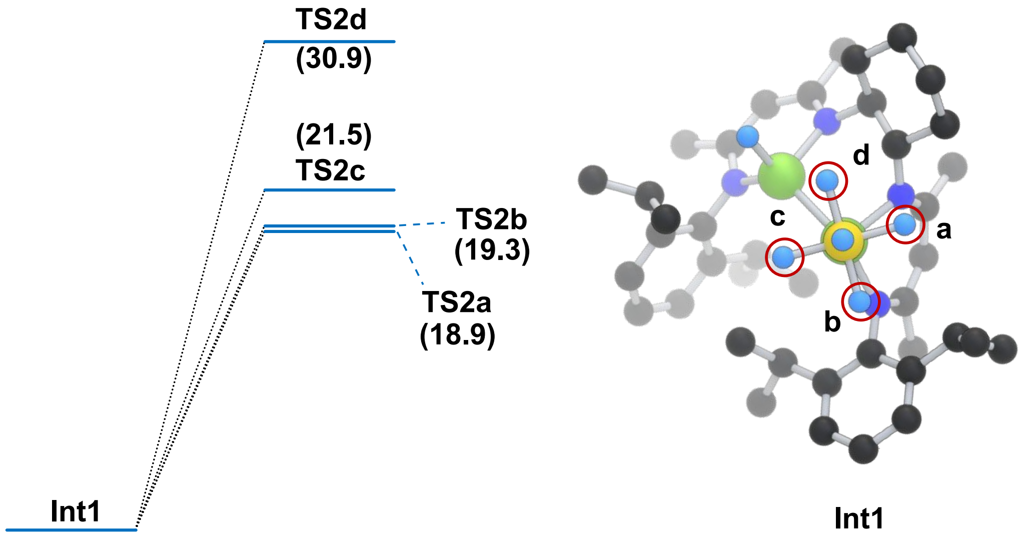

Figure S36. Activation energies of different reductive elimination sites **a – d**. All transition states were obtained through the nudged elastic band approach at the DLPNO-CCSD(T)/def2-TZVPP//M06-2X/def2-TZVP level of theory. Energies are given in kJ·mol^{-1}.

3.5. Activation Barriers of SF_x Intermediates

To investigate the influence of the SF_4 and SF_2 intermediates on the activation barrier, **TS1** and **TS3** were reoptimized at the M06-2X/def2-TZVP level of theory by sequentially removing two fluorine atoms while maintaining the key atoms involved in the transition state. The corresponding intermediates (**1a** and **Int2**) were also reoptimized in the same manner. Activation energy barriers were then evaluated at the DLPNO-CCSD(T)/def2-TZVPP level of theory, with Gibbs free energy corrections derived from the preceding DFT calculations at either 298.15K or 353.15K.

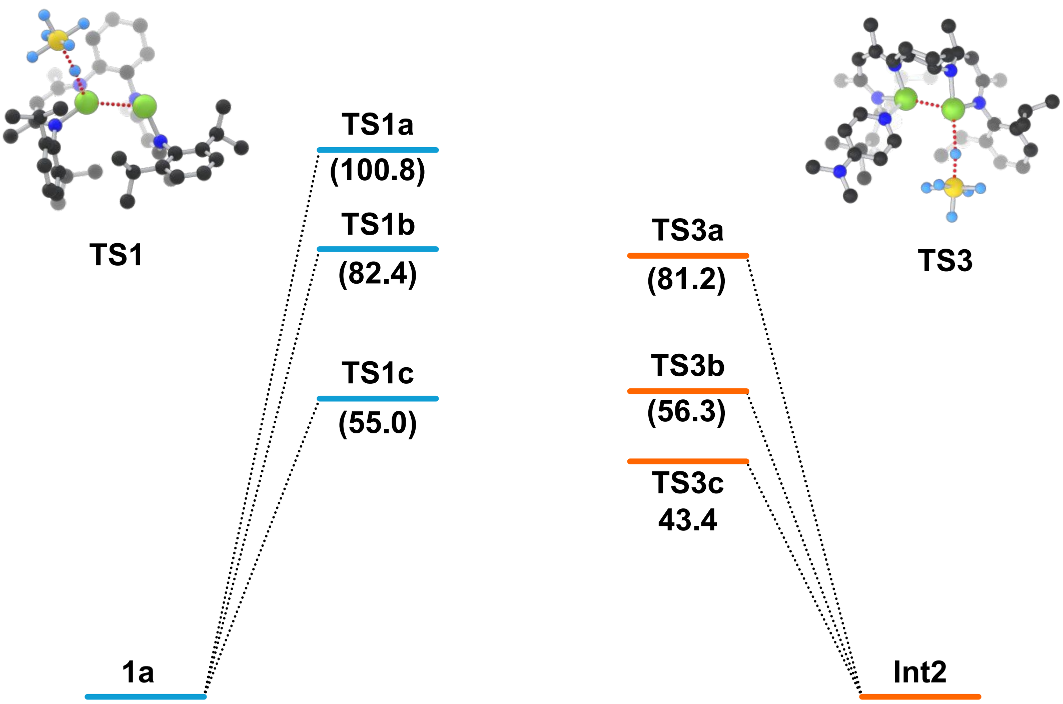

Figure S37. Activation barriers of the rate-limiting step from both uncatalyzed (**TS1** left) and DMAP facilitated (**TS3** right) pathways with (a) SF_6 (b) SF_4 and (c) SF_2 intermediates. All calculations were performed at the DLPNO-CCSD(T)/def2-TZVPP//M06-2X/def2-TZVP level of theory. Energies are given in $kJ{\cdot}mol^{-1}$.

4. References

S1 Bruker AXS **2001**, *Apex3 and SADABS*, Bruker AXS Inc., Madison, Wisconsin, USA.

S2 G. M. Sheldrick, *Acta Crystallogr.* **2015**, *A71*, 3–8.

S3 G. M. Sheldrick, *Acta Crystallogr.* **2015**, *C71*, 3–8.

S4 O. V. Dolomanov, L. J. Bourhis, R. J. Gildea, J. A. K. Howard, H. Puschmann, *J Appl Crystallogr* **2009**, *42*, 339.

S5 A. Seifert, D. Scheid, G. Linti, T. Zessin, *Chem. Eur. J.* **2009**, *15*, 12114.

S6 K. Hori, H. Motohashi, D. Saito K. Mikami, *ACS Catal.* **2019**, *9*, 417.

S7 D. Polterauer, P. Hanselmann, R. Littich, M. Bersier, D. M. Roberge, S. Wagschal, C. A. Hone, C. O. Kappe, *Org. Process Res. Dev.* **2023**, *27*, 2385.

S8 N. J. Hardman, B. E. Eichler, P. P. Power, *Chem. Commun.* **2000**, 1991.

S9 S. Singh, H.-J. Ahn, A. Stasch, V. Jancik, H. W. Roesky, A. Pal, M. Biadene, R. Herbst-Irmer, M. Noltemeyer, H.-G. Schmidt, *Inorg. Chem.* **2006**, *45*, 1853.

S10 Open Chemistry Project. openchemistry.org.

S11 Y. Zhao, D. G. Truhlar, *Theor Chem Account*, **2008**, 120, 215–241.

S12 E. Van Lenthe, E. J. Baerends, *Inc. J Comput Chem*, **2003**, 24, 1142–1156.

S13 AMS. scm.com.

S14 K. Kitaura, K. Morokuma, *Int. J. Quantum Chem*, **1976**, 10, 2, 325-340.

S15 T. Ziegler, A. Rauk, *Inorg. Chem.*, **1979**, 18, 7, 1755–1759.

S16 Ziegler, A. Rauk, *Inorg. Chem.*, **1979**, 18, 6, 1558–1565.
